# Reduced vaccine-induced germinal center outputs in patients with inflammatory bowel disease treated with anti-TNF biologics

**DOI:** 10.1172/JCI192589

**Published:** 2025-07-29

**Authors:** Michelle W. Cheung, Samantha Xu, Janna R. Shapiro, Freda Qi, Melanie Delgado-Brand, Karen Colwill, Roya M. Dayam, Ying Liu, Jenny D. Lee, Joanne M. Stempak, James M. Rini, Vinod Chandran, Mark S. Silverberg, Anne-Claude Gingras, Tania H. Watts

**Affiliations:** 1 Department of Immunology, University of Toronto, Toronto, Ontario, Canada.; 2 Lunenfeld-Tanenbaum Research Institute at Mount Sinai Hospital, Sinai Health System, Toronto, Ontario, Canada.; 3 Department of Molecular Genetics, University of Toronto, Toronto, Ontario, Canada.; 4 Zane Cohen Centre for Digestive Diseases, Division of Gastroenterology, Mount Sinai Hospital, Sinai Health System, Toronto, Ontario, Canada.; 5 Department of Biochemistry, University of Toronto, Toronto, Ontario, Canada.; 6 Gladman Krembil Psoriatic Arthritis Research Program, Schroeder Arthritis Institute, Krembil Research Institute, University Health Network, Toronto, Ontario, Canada.; 7 Division of Rheumatology, Department of Medicine,; 8 Department of Laboratory Medicine and Pathobiology,; 9 Institute of Medical Science, and; 10 Division of Gastroenterology, Department of Medicine, University of Toronto, Toronto, Ontario, Canada.

**Keywords:** Immunology, Infectious disease, Adaptive immunity, Vaccines

## Abstract

**BACKGROUND:**

Anti-TNF biologics are widely used to treat patients with immune-mediated inflammatory diseases. In mouse models, the complete absence of TNF impairs germinal center (GC) responses. Less is known about the impact of anti-TNF therapy on specific immune responses in humans. Widespread vaccination against SARS-CoV-2 offered an unprecedented opportunity to investigate the effects of biological therapies on responses to specific immunization. Previous work demonstrated that patients with inflammatory bowel disease (IBD) who were treated with anti-TNF biologics exhibited decreased Spike-specific antibody responses compared with patients with IBD treated with anti-IL-12/23 or healthy controls, even after 4 doses of mRNA vaccine.

**METHODS:**

Here we analyzed humoral responses to SARS-CoV-2 immunization using single-cell RNA-Sequencing and flow cytometry of Spike-specific memory B cells (MBC), as well as avidity measurements of plasma antibodies from patients with IBD treated with anti-TNF or anti-IL-12/23 and from people in the healthy control group.

**RESULTS:**

We observed decreased somatic hypermutation in the B cell receptors of Spike-specific MBCs and decreased antigen-specific MBC accumulation following SARS-CoV-2 mRNA vaccination in patients with IBD treated with anti-TNF, compared with patients with IBD treated with anti-IL-12/23 or people in the healthy control group. This decreased somatic hypermutation in Spike-specific MBCs in patients treated with anti-TNF correlated with decreased and delayed antibody affinity maturation and reduced neutralization activity.

**CONCLUSION:**

These data provide in vivo evidence that anti-TNF, but not anti-IL-12/23, therapy impairs the quantity and quality of antigen-specific GC outputs in humans.

**FUNDING:**

Juan and Stefania Speck (donation) and by Canadian Institutes of Health Research (CIHR)/COVID-Immunity Task Force (CITF) grants VR-1 172711, VS1-175545, GA2-177716, GA1-177703 and CIHR FDN 143301 &143350.

## Abstract 2

In depth analysis reveals profound effects of maintainence therapy of anti-TNF biologics on the affinty maturation of the antibody response and memory responses in response to vaccination, compared to anti-IL-12/23 treatment.

## Graphical abstract

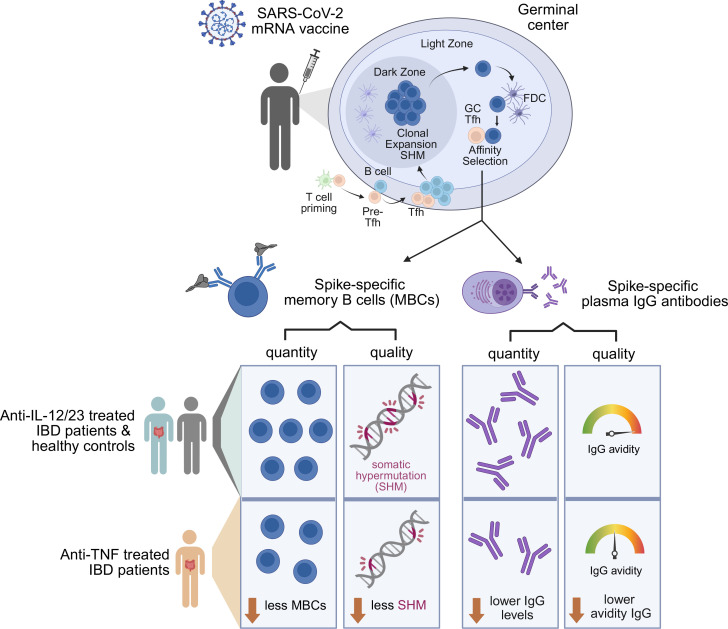

## Introduction

Immune-mediated inflammatory diseases (IMIDs) comprise a large group of heterogenous, chronic, and disabling conditions that involve one or more organs and share common inflammatory pathways. The estimated prevalence of IMIDs in North America and Europe is 5%–7%, and, in 2019, the global incidence of IMIDs was approximately 67.5 million cases (1, 2). Anticytokine biologics, such as TNF-blocking Abs, are among the most widely used therapeutics for the treatment of these conditions (3, 4).

Patients with IMID have greater susceptibility to viral infections (5, 6). However, few studies have tested the effect of cytokine blockade on a specific immune response in these patients (7). The prioritization of patients with IMID for early vaccination against SARS-CoV-2 in Canada provided an opportunity to study in detail how anticytokine biologics impact the response to a specific immunization. The IMPACT study (IMmune resPonse After COVID-19 vaccination during maintenance Therapy in IMIDs) investigated the immune responses to SARS-CoV-2 mRNA vaccination in a cohort of uninfected patients with IMID treated with antimetabolites or anticytokine biologics, from prevaccination to up to 4 doses of vaccine (8, 9). While patients with IMID overall showed decreased T cell responses after 2 doses of vaccine compared with people in the healthy control group, these differences were largely eliminated after a third dose (8, 9). However, Ab responses revealed striking differences between treatment groups, such that anti-TNF–treated patients with inflammatory bowel disease (IBD) had decreased Spike-specific (S-specific) Ab responses even after 4 doses of vaccine, compared with people in the healthy control group and patients with IBD who were treated with other therapies (8). These findings agree with other studies reporting impaired vaccine induced humoral responses in patients with IMID treated with anti-TNF in the context of SARS-CoV-2 (10–15), influenza (16–18), and hepatitis B vaccines (19).

The mechanism by which anti-TNF Abs impair the humoral response to vaccination in humans remains incompletely understood. TNF is a potent proinflammatory cytokine with pleiotropic effects on the immune system and is critical for the proper organization of germinal centers (GCs) within lymphoid organs (20, 21). In GCs, B cells acquire somatic hypermutations (SHM) in their antigen specific B cell receptors (BCRs). These mutated B cells compete for antigen, and those with the highest affinity receptors undergo preferential clonal expansion, leading to affinity maturation of the B cell response (22). This iterative process requires the complex interplay between B cells, T follicular helper cells (Tfh) and follicular dendritic cells (FDCs) (22). Selected GC B cells differentiate into memory B cells (MBCs) and long-lived Ab-secreting plasma cells (LLPCs), with the LLPCs emerging later in the GC response and enriched in cells that secrete high affinity Abs (23). Most research on the role of TNF in GCs has been conducted in murine models, where B cell–derived TNF and lymphotoxin β are required for the development of mature splenic FDC networks (24). TNF-and TNFR1-deficient mice cannot form splenic primary B cell follicles nor organized FDC networks, and, upon immunization with T cell-dependent antigens, fail to generate GCs in peripheral lymphoid organs, resulting in impaired IgG responses (20, 21). In humans, treatment of patients with rheumatoid arthritis (RA) with the TNFR2 decoy receptor etanercept led to reduced peripheral MBCs frequencies, diminished FDC networks in tonsils, and reduced GC counts and sizes compared with methotrexate-treated patients with RA and people in the healthy control group (25). Together, these findings suggest that TNF blockade disrupts GCs, in part by affecting FDC networks and the formation and maintenance of GCs. However, the precise impact of this treatment on GC outputs during a specific immune response in humans remains poorly understood.

The availability of longitudinal human blood samples from SARS-CoV-2–vaccinated yet uninfected patients with IMID provides an unprecedented opportunity to investigate the effects of cytokine blockade on a specific immune response. To this end, we isolated SARS-CoV-2 S-specific MBCs from the blood of vaccinated anti-TNF or anti-IL-12/23-treated patients with IBD as well as from vaccinated people in the healthy control group. We demonstrate that anti-TNF Ab–treated patients with IBD exhibit altered GC outputs in response to SARS-CoV-2 mRNA vaccination, characterized by reduced SHM and a lower frequency of S-specific MBCs. These findings correlated with lower levels of S-specific IgG and reduced and delayed affinity maturation of the Ab response. In contrast, the GC outputs of anti-IL-12/23–treated patients with IBD were indistinguishable from people in the healthy control group. Our findings are one of the few in-human studies demonstrating the effect of TNF blockade on a specific immune response.

## Results

### Study design and participant characteristics

**Figure 1:**
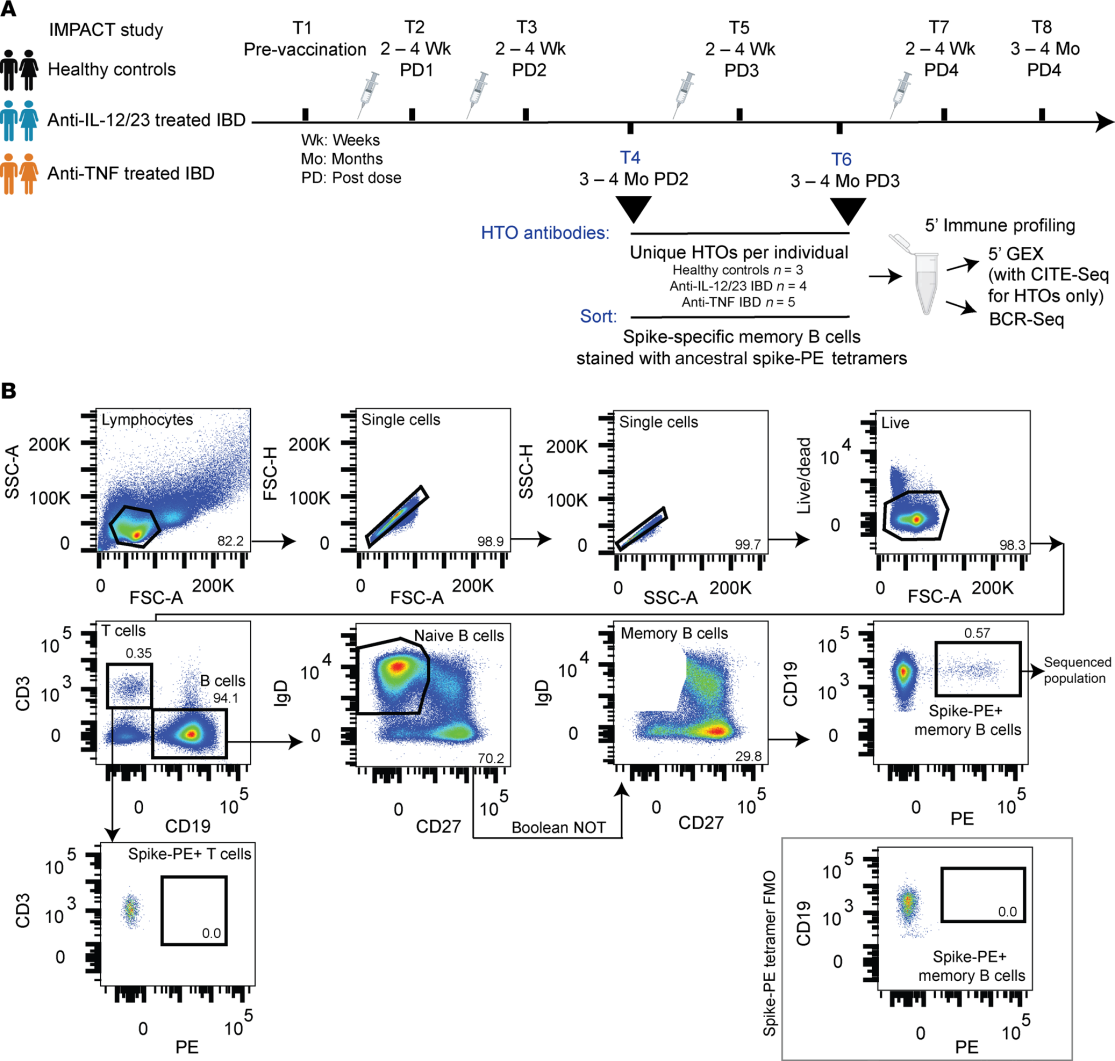
Study design and isolation of S-specific memory B cells for single-cell RNA-Seq. (**A**) Study design. Blood was collected from healthy controls, patients with IBD treated with anti-IL-12/23 and patients with IBD treated with anti-TNF at timepoints (T) before and after vaccination and 1 to 4 vaccine doses. From a subset of individuals (healthy controls, *n =* 3; anti-IL-12/23 treated IBD patients, *n =* 4; anti-TNF treated IBD patients, *n =* 5), B cell–enriched samples (following negative selection) from 3–4 months after dose 2 and after dose 3 were tagged with unique TotalSeq-C Hashtag oligo-tagged (HTO) Abs and ancestral SARS-CoV-2 S-specific (Spike-PE^+^) memory B cells were sorted for single-cell RNA-seq (5’ gene expression with CITE-Seq for hashtags) and BCR-Seq. (**B**) Representative sorting strategy for S-PE tetramer bound (Spike-PE^+^) memory B cells, gating on lymphocytes → singlets → live → CD19^+^ B cells → memory B cells (not naive B cells) → sorting for Spike-PE^+^ memory B cells. The absence of nonspecific binding of tetramer can be observed on CD3^+^ T cells. A Spike-PE tetramer FMO sample is displayed in the gray box.

This study used residual stored PBMC and plasma from the IMPACT study (8, 9). Samples were selected from patients with IMID diagnosed with IBD (ulcerative colitis or Crohn’s disease) on maintenance treatment with anti-TNF or anti-IL-12/23 biologics. As anti-TNF and anti-IL-12/23 treated patients with IBD had broadly similar T cell responses to SARS-CoV-2 vaccination, but differed in their humoral responses, the anti-IL-12/23–treated patients with IBD along with healthy donors served as controls to assess the impact of anti-TNF on GC outputs to vaccination (8, 9). The anti-TNF Abs included infliximab, adalimumab, or golimumab; however, only samples from the patients on infliximab were used for single-cell RNA-Seq. The anti-IL-12/23 biologic used was ustekinumab. Except for 1 patient who received 3 mg/day of the corticosteroid Entocort, all patients in this study were treated with a single immunomodulatory drug. The majority (at least 75% at each time point) of the patients with IBD were in remission and all were on maintenance treatment throughout the study (Supplemental Tables 1–3; supplemental material available online with this article; https://doi.org/10.1172/JCI192589DS1). None of the participants used in this analysis had been infected with SARS-CoV-2 at the time of sampling, based on testing seronegative for nucleocapsid protein as well as on self reporting. Figure 1A shows the vaccination and sample collection schedule for this study. MBCs for single-cell RNA-Seq were isolated from samples taken at 3–4 months after the second and third vaccine doses, to allow time for MBC responses to develop (Figure 1B). Table 1 and Supplemental Tables 1–3 provide in-depth demographics and sample sizes for each readout. Ethnicity data were not collected for these patients.

### Altered proportions of S-specific MBC subsets in patients with IBD treated with anti-TNF

To identify S-specific MBCs for sequencing analysis, we generated S-Streptavidin Phycoerythrin (S-PE) tetramers corresponding to the original ancestral strain of SARS-CoV-2 used in the vaccine. The specificity of S-PE tetramer binding was validated using PBMCs from prevaccination and postvaccination healthy donors (Supplemental Figure 1). MBCs were defined based on the expression of IgD and CD27, thereby excluding naive B cells (IgD^+^CD27^–^) (Supplemental Figure 1A). We observed some S-PE tetramer background binding to naive B cells in both pre- and postvaccination samples; however, as naive B cells were excluded from subsequent analysis, this was not a concern. Importantly, there was minimal S-PE tetramer binding to MBCs obtained from prevaccination samples and the frequency of S-PE^+^ MBCs increased approximately 10-fold in postvaccination samples (Supplemental Figure 1B), confirming that the S-PE tetramer binding was specific.

**Figure 2:**
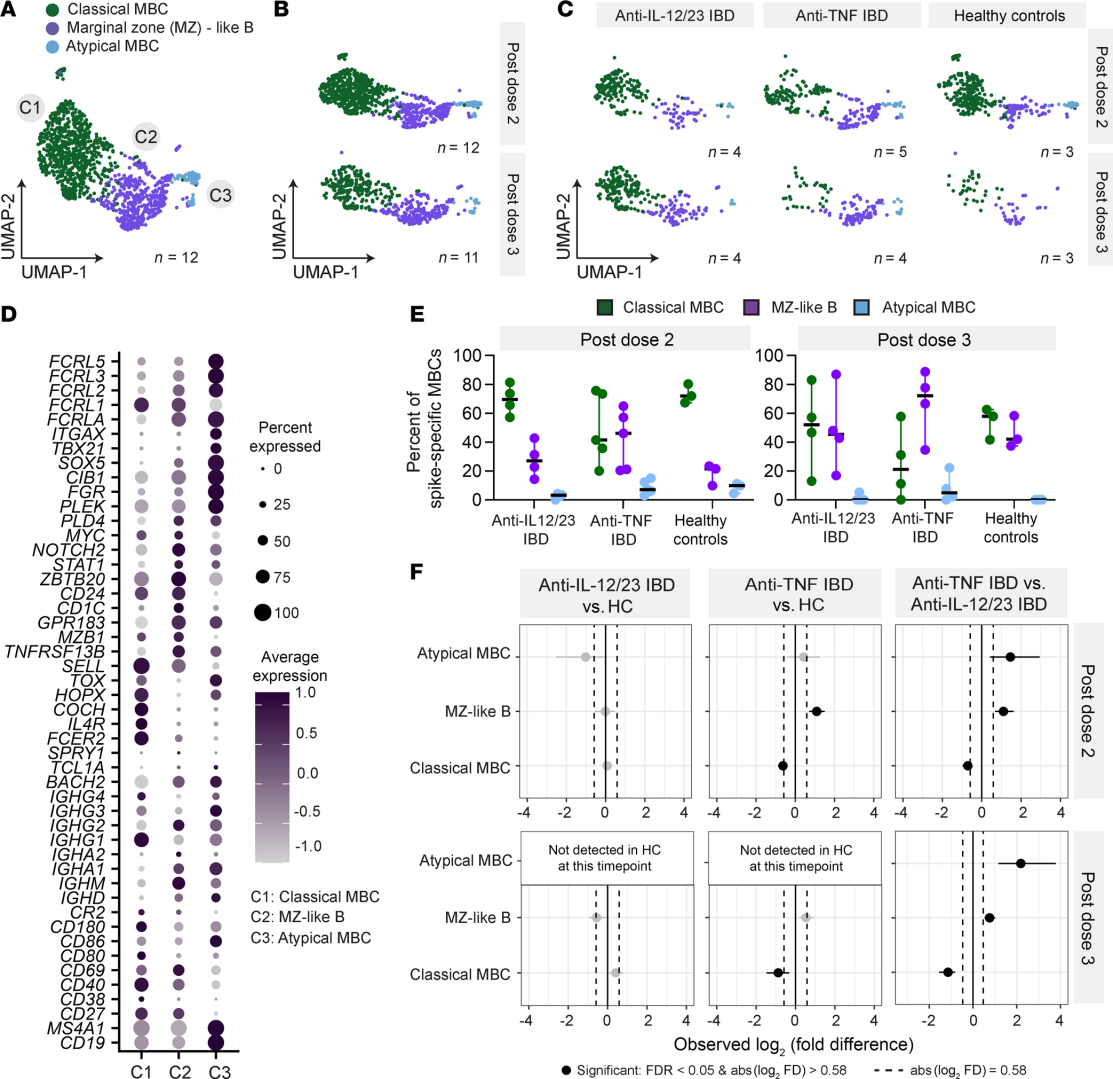
Transcriptomic analysis reveals altered proportions of S-specific memory B cell subsets in IBD patients treated with anti-TNF. (**A**) UMAP of S-specific memory B cells (MBC). Clusters are colored by subtype: C1, classical MBC; C2, marginal zone–like (MZ) B cells; C3, atypical MBC. Total number of individuals: *n =* 12 (healthy controls *n =* 3, patients with IBD treated with anti-TNF *n =* 5, patients with IBD treated with anti-IL-12/23 *n =* 4); total cell count: *n =* 1,268. (**B**) UMAP of S-specific MBCs separated by timepoint: 3–4 months after dose 2 (*n* cells = 824) or 3–4 months post dose 3 (*n* cells = 444). (**C**) UMAP of S-specific MBCs separated by timepoint and by study group: MBC after vaccine dose 2 in patients with IBD treated with anti-IL-12/23 (*n* cells = 215); MBC after vaccine dose 3 in patients with IBD treated with anti-IL-12/23 (*n* cells = 256); MBC after vaccine dose 2 in IBD patients treated with anti-TNF (*n* cells = 252); MBC after vaccine dose 3 in patients with IBD treated with anti-TNF (*n* cells = 125); MBC after vaccine dose 2 in the healthy control group (*n* cells = 357); MBC after vaccine dose 3 in the healthy control group (*n* cells = 63). (**D**) DotPlot depicting the expression of selected genes. Dot size corresponds to the percentage of cells expressing the gene. Color intensity of the dots corresponds to the average expression across cells, where “0.0” represents the mean expression across the whole dataset. (**E**) Percentage of S-specific MBCs of each subset. Each dot represents one individual. Data represent median ± 95% CI. (**F**) Permutation tests were utilized to compare the proportion of S-specific MBCs classified as each MBC subset between study groups. The values colored in grey are nonsignificant; values colored in black are significant (FDR < 0.05 & absolute value of log_2_(fold difference) > 0.58. (**A**–**F**) Anti-IL-12/23 IBD, patients with IBD treated with anti-IL-12/23; anti-TNF IBD, patients with IBD treated with anti-TNF.

We performed 5’ immune profiling via single-cell RNA-Seq and BCR-Seq on S-PE–binding MBCs sorted from the B-cell enriched PBMCs of 3 people in the healthy control group, 5 patients with IBD treated with anti-TNF, and 4 patients with IBD treated with anti-IL-12/23 (Table 1), using matched samples from 3–4 months after dose 2 and from 3–4 months after dose 3 (Figure 1). A total of 1,268 cells were analyzed after quality control (824 cells after dose 2 and 444 cells after dose 3, with cell numbers limited by available samples) (Figure 2A). Cells were clustered based on transcriptional data and visualized by uniform manifold approximation and projection (UMAP). Initial identification of major cell clusters was based on well-known cell-specific markers — naive B cells (high *IGHD, IGHM, TCL1A,* and *BACH2* expression and lack of the B cell memory marker *CD27*); memory B cells (high *CD27,* low/absent *IGHD* and *CD38*); and NK cells (lack *CD19* and *MS4A1* [CD20] expression but express *GNLY* and *NKG7*). Contaminating naive B cells and NK cells were excluded from downstream analyses and cells were reclustered to identify MBC subsets (Figure 2A). Labeling of individual samples with TotalSeq-C hashtag oligo–tagged Abs enabled the demultiplexing of samples by timepoint (Figure 2B), study group (Figure 2C), and individual (Supplemental Figure 2).

Circulating MBCs in human blood can be broadly divided into 2 subsets. Classical MBCs (cMBCs) derive from germinal centers, express CD27 and CD21, but lack the expression of CD11c, while atypical MBCs (aMBCs) also referred to as double negative (DN) 2 B cells or age-associated B cells, lack CD21 and CD27 and express CD11c (26, 27). Marginal zone (MZ) B cells are innate-like B cells that predominantly reside in the splenic MZ, but can also be found in lymph nodes and blood and are largely unswitched, IgM expressing cells (28). In our analyses of S-specific MBCs, we identified 3 clusters with distinct surface marker and transcriptomic profiles: cMBCs (cluster 1; C1), MZ-like B cells (cluster 2; C2), and aMBCs (cluster 3; C3) (Figure 2D and Supplemental Figure 3). Classical MBCs were identified as CD27^+^ IgD^–^ and were predominantly class switched, expressing the 4 *IGHG* subtypes (Figure 2D, Supplemental Figure 3A). Differential gene expression analyses revealed robust expression of *TOX, HOPX,* and *COCH* in cMBCs (Figure 2D and Supplemental Figure 3B), as previously reported (29). Classical MBCs also showed increased *IL-4R, IL-13RA1*, *FCER2* (encoding CD23), and *HLA-DMB,* consistent with increased T cell–derived IL-4 responsiveness and a GC origin of cMBCs (Supplemental Figure 3B) (30). Cluster 2, which we defined as “MZ-like B cells,” contained the greatest proportion of unswitched cells expressing *IGHM* and a lower proportion of cells expressing switched isotype genes (Figure 2D and Supplemental Figure 3A). Furthermore, we observed higher levels of genes associated with the MZ B cell phenotype: *CD24, PLD4*, *MZB1, CD1c*, *FCGR2B*, *TNFRSF13B*, and *GPR183* (EBI2) (Figure 2D and Supplemental Figure 3B) (31–35). *AKT3* and *PTPRJ* (CD148) were upregulated in the MZ-like B cluster (Supplemental Figure 3B), also consistent with the literature (36, 37). Finally, we detected a small population of MBCs lacking CD27 and expressing genes associated with an atypical phenotype, such as *ITGAX*, encoding surface protein CD11c, *TBX21*, *TOX*, *SOX5,* Fc-receptor-like genes *FCRLA, FCRL3* and *FCRL5, FGR,* inhibitory receptor *CD72*, and high *CD19* and *CD20* expression (Figure 2D and Supplemental Figure 3, A and B) (38).

To identify whether the transcriptome of S-specific cMBCs and MZ-like B cells differed between study groups, we conducted pairwise differential gene expression analyses between study groups (Supplemental Figure 4). Atypical MBCs were excluded from the analyses due to their low cell count. Overall, few transcripts were different between groups. The increased Y chromosome–specific transcripts (*DDX3Y, KDM5D*, *RPS4Y1, TXLNGY, UTY, UPS9Y)* in the anti-TNF–treated IBD study group compared with the anti-IL-12/23–treated IBD and healthy control groups likely reflects differences in the male-to-female ratio in the study groups analyzed (Table 1). We also observed increased MHC class II gene expression in the cMBCs (*HLA-DQA2*, *HLA-DRB6*) (Supplemental Figure 4A) and MZ-like B cells (*HLA-DQA2*, *HLA-DRB5*) (Supplemental Figure 4B) of patients with IBD (anti-IL-12/23 or anti-TNF treated) compared with people in the healthy control group. However, we did not observe an increased overall MHC class II expression on total and S-specific cMBCs and MZ-like B cells at the protein level (Supplemental Figure 4, C and D). *RPS26* expression was increased in patients with IBD compared with people in the healthy control group. RPS26 has been positively correlated with anemia, which is a common complication in patients with IBD, and identified as a differentially expressed gene marker of IBD (39, 40).

To assess quantitative differences in each cluster between people in the healthy control group and patients with IBD who have been treated, we calculated the percentage of S-specific MBCs in each of the 3 MBC subsets, normalized to each individual (Figure 2E). For the anti-IL-12/23–treated patients with IBD and people in the healthy control group, most S-specific MBCs were classified as cMBCs, followed by MZ-like B cells and a small fraction of aMBCs (Figure 2E). Atypical MBCs were not detected in people in the healthy control group after dose 3, likely due to the low overall counts of S-specific aMBCs isolated rather than a biological effect (Figure 2E). Patients who were treated with anti-TNF tended to have similar or higher percentages of S-specific MZ-like B cells compared with cMBCs (Figure 2E). The anti-TNF–treated group had significantly higher proportions of S-specific MZ-like B cells and aMBCs, and lower proportions of cMBCs after dose 2 and 3, compared with people in the healthy control group and patients with IBD treated with anti-IL12/23 (Figure 2F), based on pairwise permutation tests with bootstrapping using the scProportionTest library in R (41).

### Patients with IBD treated with anti-TNF have reduced frequencies of S-specific cMBC

**Figure 3:**
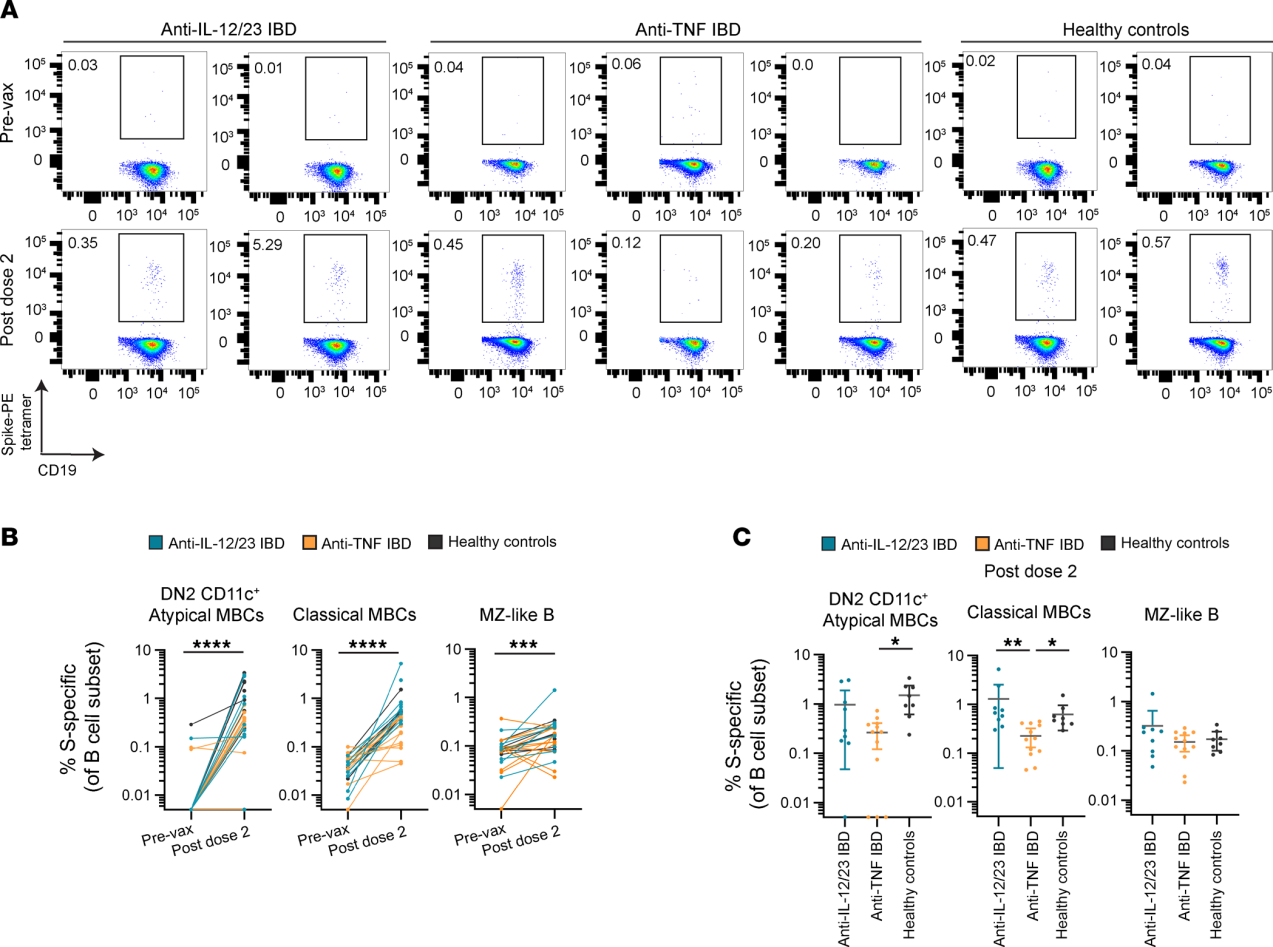
Reduced S-specific classical memory B cells in patients with IBD treated with anti-TNF. (**A**) Representative flow cytometric plots of S-specific classical memory B cells (MBC) from patients with IBD treated with anti-TNF (*n =* 2), patients with IBD treated with anti-IL-12/23 (*n =* 3) and people in the healthy control group (*n =* 3) at prevaccination and 3–4 months after dose 2. The frequency (%) of S-PE tetramer positive B cells is shown as a percentage of classical MBCs (CD19^+^ IgD^–^CD27^+^). (**B**) Frequency of S-specific MBCs prevaccination and 3–4 months after dose 2, grouped by MBC subset. Wilcoxon matched pairs signed rank 2-tailed tests were performed. (**C**) Frequency of S-specific MBCs at 3–4 months after dose 2, grouped by MBC subset. Kruskal-Wallis 1-way ANOVA with Dunn’s multiple comparisons tests were performed. (**B** and **C**) Patients with IBD treated with Anti-IL-12/23 (*n =* 9) are colored teal, patients with IBD treated with anti-TNF (*n =* 12) are colored orange, and people in the healthy control group (*n =* 8) are colored black. B cell subsets: DN2 (double negative) CD11c^+^ atypical MBCs, classical MBCs and marginal zone-like (MZ) B cells. *P < 0.05; **P < 0.01; ***P <0.001; ****P <0.0001. Data represent mean ± 95% CI. Sample size information is provided in Supplemental Table 1. (**A**–**C**) Anti-IL-12/23 IBD, patients with IBD treated with anti-IL-12/23; anti-TNF IBD, patients with IBD treated with anti-TNF.

To validate the differences in MBC subsets in the anti-TNF treated patients observed in the RNA-Seq data, we assessed the phenotype of total peripheral blood B cells and S-specific MBCs in the 3 study groups using flow cytometry (Figure 3 and Supplemental Figure 5). These samples were from different individuals than those used for sequencing, thereby serving as a validation cohort (Supplemental Table 1). MBCs in PBMCs collected prevaccination and 3–4 months after dose 2 were subtyped based on surface markers and Ig isotype as either MZ-like B cells (CD27^+^IgD^+^), cMBCs (CD27^+^IgD^–^), or DN B cells (CD27^–^IgD^–^). DN B cell subsets were further defined as DN1 (CD21^+^CD11c^–^), DN2 (CD21^–^CD11c^+^) CD11c^+^ aMBCs, or DN3 (CD21^–^CD11c^–^) (Supplemental Figure 5A) (42). Compared with cMBCs, MZ-like B cells lacked CD23 expression and highly expressed CD21, IgM, and CD1c, while CD11c^+^ aMBCs lacked the expression of CD21 and CD27 and had high levels of CD20 and MHC class II (pan-HLA-DR/DP/DQ) (Supplemental Figure 5B), as previously reported (43, 44).

There were no significant differences in the frequencies of DN B cells, cMBCs, MZ-like B cells or naive B cells among total peripheral blood B cells between study groups prevaccination or after dose 2 (Supplemental Figure 5C). However, people in the healthy control group exhibited higher frequencies of DN1 B cells and lower frequencies of DN3 B cells compared with patients with IBD treated with anti-TNF (Supplemental Figure 5D). There was also no difference in the percentage of unswitched IgM^+^ cMBCs or switched IgG^+^ or IgG^–^IgM^–^ cMBCs between study groups (Supplemental Figure 5E).

We used S-PE tetramers to determine the frequencies of S-specific MBCs within each subset of MBC at prevaccination and 3–4 months after dose 2 (Figure 3, A–C). Prevaccination, there were minimal S-specific aMBCs and cMBCs, as expected; however, there was a small population of S-specific MZ-B cells (Figure 3B). Significant increases in the percentage of S-specific MBCs relative to total B cells within each MBC subset were observed after dose 2 (Figure 3B). Strikingly, after 2 vaccine doses, patients with IBD treated with anti-TNF demonstrated significantly lower frequencies of S-specific cMBCs compared with patients with IBD treated with anti-IL-12/23 and lower frequencies of S-specific DN2 CD11c^+^ aMBCs and cMBCs compared with people in the healthy control group (Figure 3, B and C). There were no differences in the frequencies of S-specific MZ-like B cells between groups (Figure 3, B and C). These data demonstrate a decreased frequency of antigen-specific MBCs in patients treated with anti-TNF; in contrast, the relative frequencies of B cell subpopulations among total peripheral blood B cells were similar in all 3 study groups. Thus, anti-TNF therapy impairs the induction of S-specific cMBCs following vaccination, consistent with their decreased proportion, as evidenced by the RNA-Seq data.

### Biased BCR V_H_ and V_L_ gene usage in S-specific MBCs

**Figure 4:**
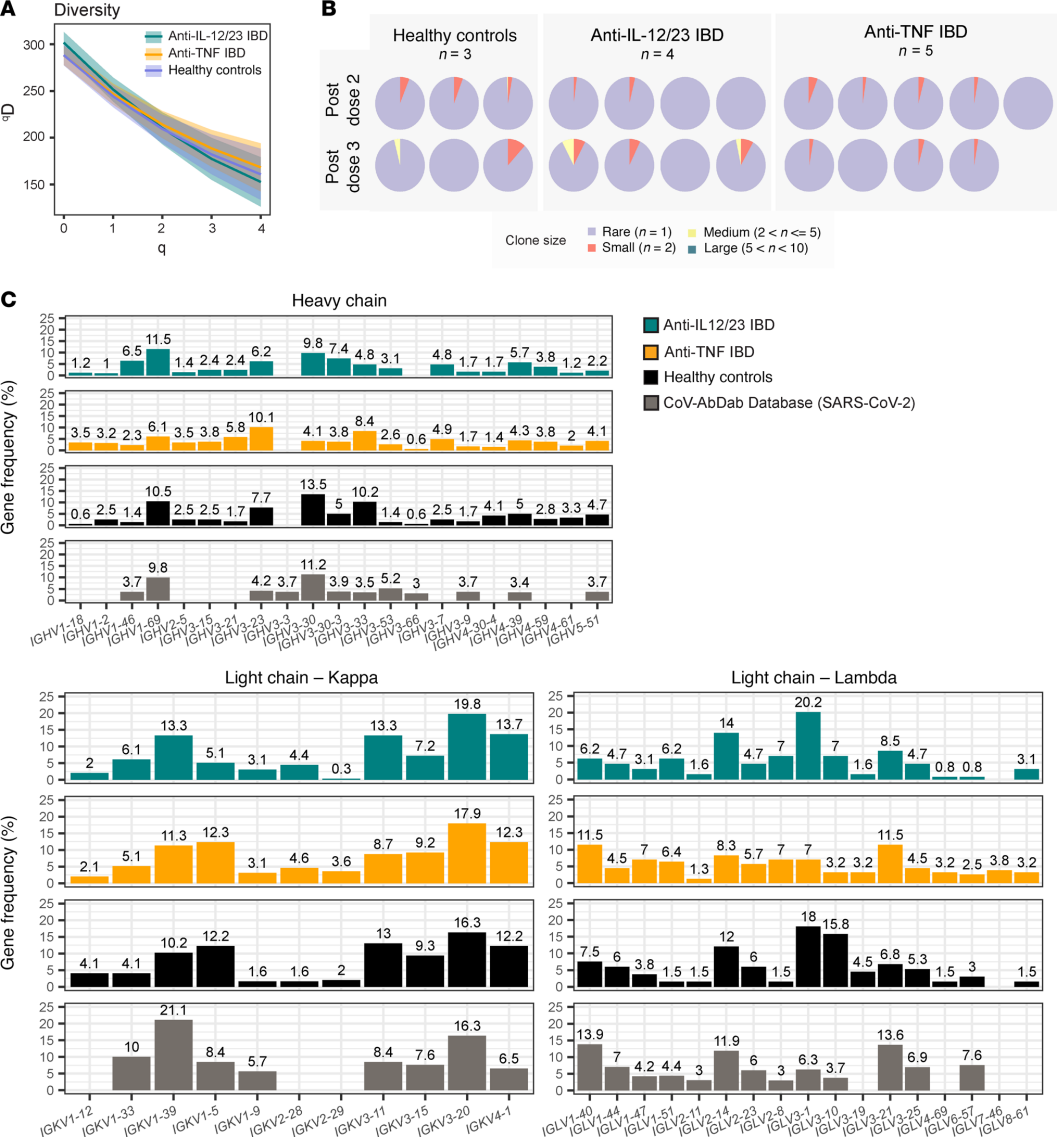
Biased BCR heavy and light chain variable gene usage in S-specific memory B cells. BCR-sequencing of people in the healthy control group (*n =* 3), patients with IBD treated with anti-IL-12/23 (*n =* 4), and patients with IBD treated with anti-TNF (*n =* 5), 3–4 months after dose 2 and 3. (**A**) Diversity (D) curve of S-specific MBCs grouped by study group (after dose 2 and dose 3 pooled). Diversity is calculated over a range of diversity orders, q (q = 1 species richness; q = 2 Shannon entropy; q = 3 Simpson’s index). Shading represents confidence intervals. (**B**) Pie charts showing MBC clonal expansion indicated for each patient and timepoint. MBCs were binned into rare clones (*n =* 1 cell), small (*n =* 2 cells), medium (2 < *n* cells ≤ 5), or large (5 < n ≤ 10). (**C**) Frequency of heavy chain variable genes and light chain κ and λ variable genes in each study group compared to the CoV-AbDab database (filtered for SARS-CoV-2 specific entries). Only genes found in at least 3% of 1 of the 4 comparison groups are plotted.

Next, we analyzed BCR sequences of S-specific MBCs after vaccine doses 2 and 3 across the 3 study groups. Clones were identified as cells sharing common heavy chain V and J genes and harboring the same complementarity determining region (CDR) 3 length. A total of 1,100 BCRs, combined from all individuals and timepoints, were analyzed (Figure 4). We quantified clonal diversity (D) over a range of diversity orders of S-specific MBCs from the 3 study groups (Figure 4A) (45). Across all metrics of diversity, high diversity was observed for patients with IBD treated with anti-IL-12/23- or anti-TNF and people in the healthy control group. We also observed high degrees of diversity when cells were stratified by individual (Figure 4B). Some clonal expansion was observed after a third dose of vaccine in people in the healthy control group and patients with IBD who were treated with anti-IL-12/23, although not in the anti-TNF–treated study group (Figure 4B).

To examine the repertoire of S-specific MBCs, we compared heavy (V_H_) and light (V_L_) chain variable gene usage across study groups (Figure 4C). Gene usage frequencies in each study group were compared with the CoV-AbDab public database of published or patented Abs reported to bind coronaviruses, which we specifically filtered for database entries that were reported to bind SARS-CoV-2 S (Figure 4C) (46). *VH3*-family genes were particularly abundant, followed by *VH1* and *VH4* families (Figure 4C). The most abundant V_H_ genes in all 3 study groups mirrored the top gene usages in the CoV-AbDab database (*IGHV1-69* and *IGHV3-30*). An enriched usage of *IGKV1-39*, *IGKV3-11*, *IGKV3-20*, and *IGLV2-14* were observed in the 3 study groups and in the CoV-AbDab database (Figure 4C). Visualization of paired V_H_ and V_L_ gene usage of S-specific MBCs is shown in Supplemental Figure 6. Of the 48 gene pairs detected as shared by the 3 study groups, an enrichment in the usage of *IGHV1-69D/IGKV3-11* was observed based on the count of S-specific MBCs detected (Supplemental Figure 7). Overall, V_H_ and V_L_ repertoire analyses revealed high diversity in each study group, as well as conserved V gene usages and V gene pairs across study groups, suggesting that the biological treatments (anti-IL-12/23 and anti-TNF Abs) had little or no impact on Ab diversity.

### Altered proportions of class-switched S-specific MBCs in patients with IBD treated with anti-TNF

**Figure 5:**
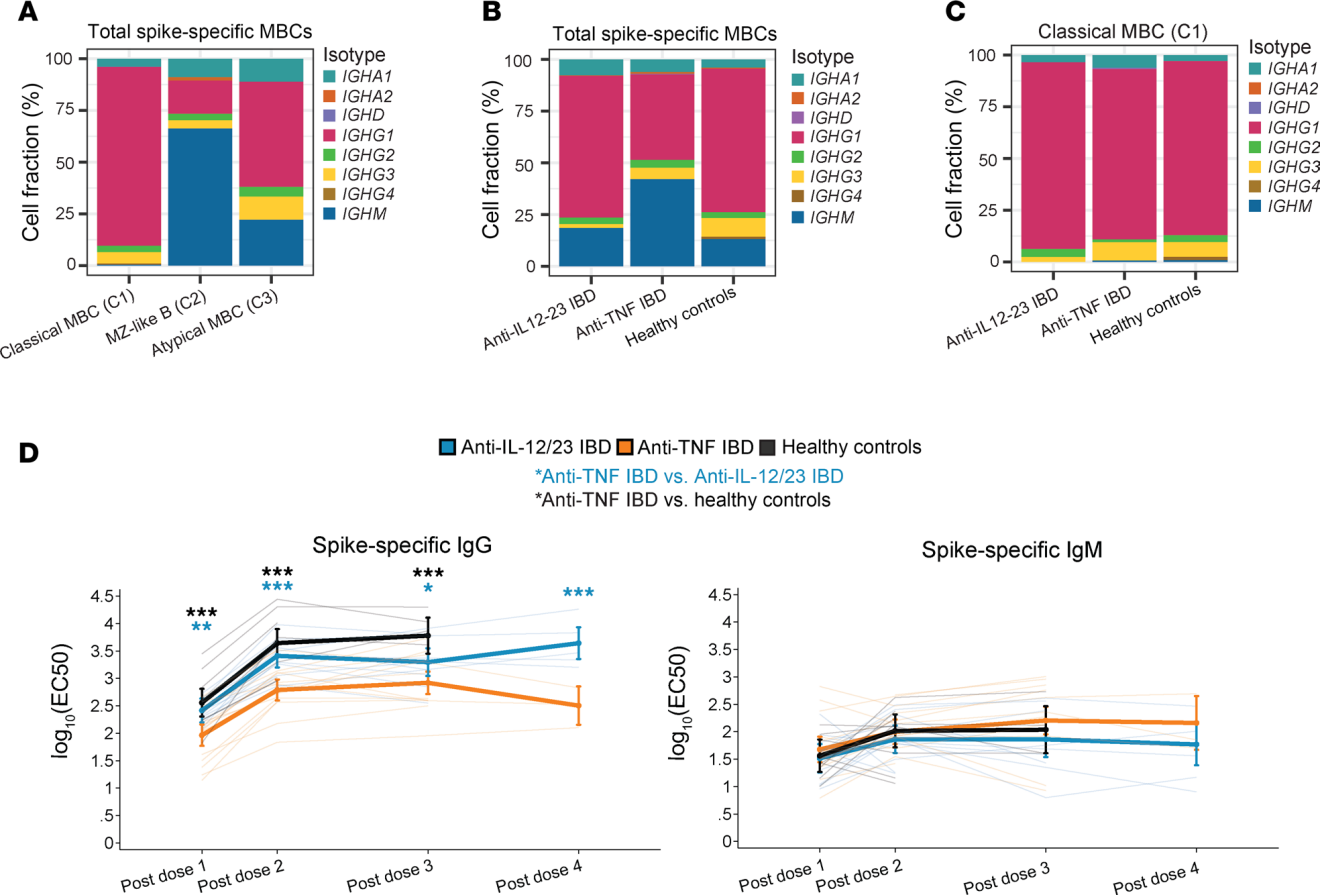
Altered proportions of switched MBCs and plasma IgG in patients with IBD treated with anti-TNF. (**A**–**C**) BCR-sequencing of S-specific MBCs 3–4 months after dose 2 and 3. C1: cluster 1; C2: cluster 2; C3: cluster 3. (**A**) Fraction (%) of total S-specific MBCs (classical MBCs, marginal zone–like (MZ) B cells, and atypical MBCs) expressing each isotype. (**B**) Fraction (%) of total S-specific MBCs (classical MBCs, marginal zone–like (MZ) B cells, and atypical MBCs) expressing each isotype, grouped by study group. (**C**) Fraction (%) of S-specific classical MBCs (cluster 1) expressing each isotype, grouped by study group. (**D**) Level of plasma S-specific IgG and IgM across timepoints 2–4 weeks after 1 to 4 vaccine doses. EC_50_, sample dilution that gives a response halfway between the minimum signal of the assay and sample’s own maximum (top) activity. *P < 0.05, **P < 0.01, ***P < 0.001, ****P < 0.0001. Asterisks in black indicate comparisons between the healthy control group and the patients with IBD treated with anti-TNF, asterisks in teal indicate comparisons between patients with IBD treated with anti-TNF and patients with IBD treated with anti-IL-12/23. Thin lines represent each individual. Multivariate regression analyses controlled for age, sex, BMI, and vaccine type, with an interaction term between timepoint and study group. Data represent mean ± 95% CI. Sample size information is provided in Supplemental Table 2. (**A**–**D**) Anti-IL-12/23 IBD, patients with IBD treated with anti-IL-12/23; anti-TNF IBD, patients with IBD treated with anti-TNF.

The BCR-seq data revealed that the cMBCs were largely class switched, expressing *IGHG1–4* or *IGHA1,* with *IGHG1* expression most predominant (Figure 5A). In contrast, approximately 75% of MZ-like B cells expressed *IGHM,* whereas approximately 25% were class switched. Atypical MBCs were heterogenous but predominantly class switched, albeit harboring a greater fraction of nonswitched *IGHM-*expressing cells than cMBCs (Figure 5A). When considering total S-specific MBCs (subsets pooled), patients with IBD treated with anti-TNF exhibited a lower proportion of MBCs expressing *IGHG1–4* class-switched BCRs, and, concomitantly, a greater proportion of BCRs expressing *IGHM* (Figure 5B). As most cMBCs expressed class-switched BCRs in all 3 study groups (Figure 5C), the lower proportion of switched MBCs in patients with IBD treated with anti-TNF overall may reflect the decreased frequency of cMBCs (Figure 2C), rather than a decrease in class switching.

### Decreased S-specific IgG, but similar S-specific IgM levels, in plasma from patients with IBD treated with anti-TNF

To investigate more directly the potential impact of anti-TNF on Ab class switching, we conducted ELISA assays for S-specific IgG and IgM from the 3 study groups. The results showed that across 4 vaccine doses, patients with IBD treated with anti-TNF had significantly reduced S-specific IgG levels, compared with patients with IBD treated with anti-IL-12/23 and people in the healthy control group (Figure 5D), consistent with our earlier studies (8, 9). In contrast, levels of S-specific IgM were similar across the 3 groups. These data show a reduced output of IgG without a proportional increase in IgM, consistent with reduced LLPC output from the GC, rather than a reduction in Ab class switching.

### Reduced somatic hypermutation in patients with IBD treated with anti-TNF

**Figure 6:**
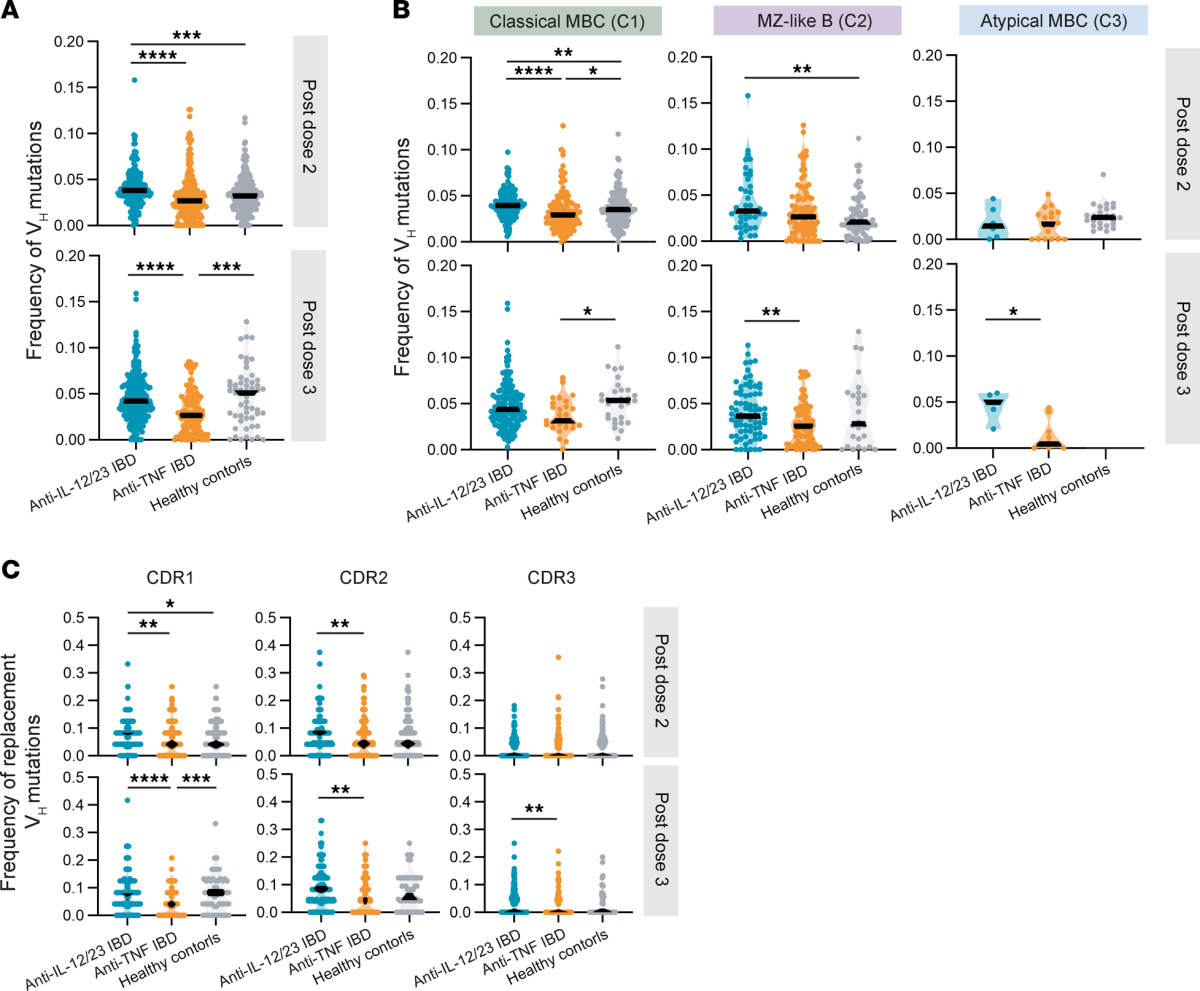
Reduced somatic hypermutation in the BCRs of S-specific memory B cells in patients with IBD treated with anti-TNF. BCR-Seq of S-specific memory B cells (MBCs) from people in the healthy control group (*n =* 3), patients with IBD treated with anti-IL-12/23 (*n =* 4), and patients with IBD treated with anti-TNF (*n =* 5), after dose 2 and 3. Each dot represents a cell. (**A**) Frequency of somatic hypermutations (SHM) in the heavy chain variable region of S-specific MBCs (classical MBCs, MZ-like B cells, and atypical MBCs pooled), after dose 2 and after dose 3, grouped by study group. (**B**) Frequency of SHM in the heavy chain variable region of S-specific MBCs after dose 2 and 3, separated by subset (classical MBCs, marginal MZ-like B cells, atypical MBCs) and grouped by study group. (**C**) Frequency of replacement SHM in the heavy chain complementarity determining regions (CDR) 1–3 of S-specific MBCs (classical MBCs, MZ-like B cells, and atypical MBCs pooled), after dose 2 and 3, grouped by study group. (**A**–**C**) Frequency is calculated as counts of mutations over the total number of positions in the V gene sequence. Kruskal-Wallis one-way ANOVA with Dunn’s multiple comparisons tests were performed. *P < 0.05, **P < 0.01, ***P < 0.001, ****P < 0.0001. Anti-IL-12/23 IBD, patients with IBD treated with anti-IL-12/23; anti-TNF IBD, patients with IBD treated with anti-TNF.

SHM in Ig variable (V) regions is important in the affinity maturation of Ab responses. Therefore, we compared the frequency of SHM in the V regions of heavy (V_H_) and light (V_L_) chains of MBCs derived from healthy controls and patients with IBD treated with anticytokines (Figure 6 and Supplemental Figure 8). Overall, cMBCs had the greatest frequencies of V_H_ and V_L_ mutations, followed by MZ-like B cells and aMBCs, irrespective of whether 2 or 3 doses of vaccine were received (Supplemental Figure 8, A and B). Stratification by study group revealed that patients with IBD treated with anti-TNF had significantly lower frequencies of V_H_ mutations in total S-specific MBCs compared to both the patients with IBD treated with anti-IL-12/23 and people in the healthy control group after the third dose of vaccine, with differences between the anti-IL-12/23 and anti-TNF groups also observed after dose 2 (Figure 6A). The greatest differences in the frequencies of V_H_ mutations were observed in the cMBCs, with the cMBCs of patients with IBD treated with anti-TNF exhibiting lower mutation frequencies compared with the other 2 groups after dose 2 (Figure 6B). Similar results were seen with V_L_ mutations, whereby patients with IBD treated with anti-TNF had a lower frequency of mutations compared with patients with IBD treated with anti-IL-12/23 and people in the healthy control group (Supplemental Figure 8C).

When SHMs in the CDRs (CDR1–3) increase the affinity of the BCR for antigen, those cells are selected for clonal expansion in the GC, resulting in affinity maturation. In contrast, mutations in structurally important positions in the framework regions are selected against (47). Therefore, we further characterized the frequency of mutations by location and the type of mutation (replacement or silent). The bulk of SHMs in the MBC BCRs in all study groups were replacement mutations concentrated in CDR1–3, with fewer SHM in the framework regions (Supplemental Figure 8D). Patients with IBD treated with anti-TNF exhibit lower frequencies of replacement V_H_ mutations in CDR1-3 compared with patients with IBD treated with anti-IL-12/23 after dose 2 and 3, and lower mutation frequencies in CDR1 compared with people in the healthy control group after dose 3 (Figure 6C). Taken together, the data show that the BCRs of S-specific MBCs from patients treated with anti-TNF exhibit less SHM, with the majority being replacement mutations, suggesting that the B cells in the anti-TNF group have undergone less efficient affinity maturation in the GC.

### Patients with IBD treated with anti-TNF have lower avidity anti-S IgG across 4 vaccine doses

**Figure 7:**
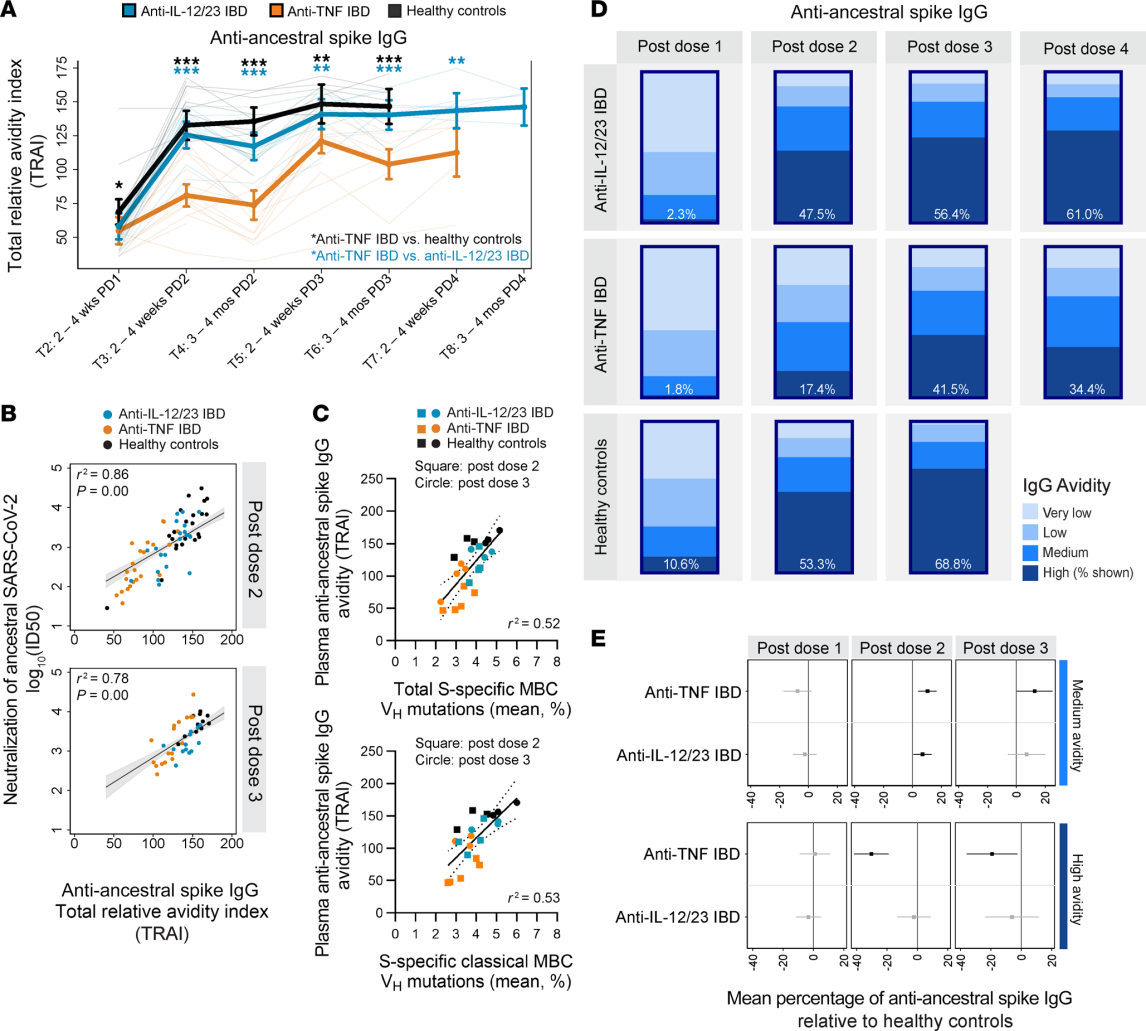
The avidity of anti-S IgG is lower in patients with IBD treated with anti-TNF across four vaccine doses. (**A**) Avidity (total relative avidity index, TRAI) of anti-ancestral S IgG across 1–4 vaccine doses. Multivariate regression analyses controlled for age, BMI, sex, vaccine type, and IgG concentration, with an interaction term between timepoint and study group. Lines represent each individual. *P < 0.05, **P < 0.01, ***P < 0.001. Colored asterisks compare patients with IBD treated with anti-TNF with people in the healthy control group (black) or with patients with IBD treated with anti-IL-12/23 (teal). (**B**) Association of anti-ancestral S IgG avidity and neutralization of ancestral SARS-CoV-2 after dose 2 and 3. Neutralization data were previously reported (8, 9). Log_10_(ID_50_), the serum dilution that inhibits 50% of lentiviral infection. Linear regressions controlled for age, BMI, sex, and vaccine type. (**C**) Association of mean frequency of heavy chain variable region somatic hypermutation in total S-specific memory B cells (MBCs) (classical MBCs, MZ-like B cells, atypical MBCs pooled) or S-specific classical MBCs and the avidity of anti-ancestral S IgG. Linear regressions were performed. Each point represents an individual. (**D**) Mean percentage of anti-ancestral S IgG that fall into each categorical avidity level: very low, low, medium, high. Percentage of high avidity IgG is indicated. Samples were obtained 2–4 weeks after each vaccine. Values are derived as fractional relative avidity indices (Supplemental Table 5). (**E**) Comparisons of the mean percentage of anti-ancestral S IgG of each categorical avidity level in treated patients with IBD relative to healthy controls, after 1–3 doses. Least squares regression controlled for age, sex, BMI, vaccine type and IgG concentration. Significant differences (*P* < 0.05) are colored black. (**A**, **D**, and **E**) Sample size and timepoint information is provided in Supplemental Table 3. (**A** and **E**) Data represent mean ± 95% CI. (**A**–**E**) Anti-IL-12/23 IBD, patients with IBD treated with anti-IL-12/23; anti-TNF IBD, patients with IBD treated with anti-TNF.

We next asked if the reduced SHM frequency in the BCRs of S-specific MBCs from patients with IBD treated with anti-TNF after vaccination was associated with a lower avidity plasma Ab response. We used a modified ELISA in which increasing concentrations of the chaotropic agent ammonium thiocyanate were added before the addition of the secondary Ab, to assess the strength of the Ab-antigen interaction (48–51). We analyzed the avidity of Ab binding to ancestral S (Figure 7), with avidity reported as a total relative avidity index (TRAI), as defined in the methods. At the time of sample collection, people in the healthy control group were not prioritized for a fourth dose of vaccine, hence data are not available beyond the third dose for the people in the healthy control group. Likewise, we did not have sufficient participants in the anti-TNF–treated IBD group to evaluate avidity at the 3–4 months after the dose 4 timepoint. In all participants, anti-S IgG avidity significantly increased with each successive dose of vaccine, plateauing after 3 vaccine doses in anti-IL-12/23–treated and anti-TNF–treated patients with IBD (Figure 7A and Supplemental Table 4). The second dose of vaccine induced the most robust increase in the avidity of S-binding IgG in the healthy control group and patients with IBD treated with anti-IL-12/23, whereas patients with IBD treated with anti-TNF exhibited the greatest boost in the avidity of S-binding IgG after the third dose (Figure 7A). Furthermore, the boost in Ig avidity after dose 3 in patients treated with anti-TNF was smaller than the boost in avidity after dose 2 in the healthy control group and patients with IBD treated with anti-IL-12/23, highlighting delayed and decreased affinity maturation (Figure 7A). Strikingly, across 1–4 doses of vaccine, patients with IBD treated with anti-TNF showed significantly lower avidity IgG binding to S compared with people in the healthy control group and patients with IBD treated with IL-12/23 (Figure 7A), consistent with the BCR-Seq data presented above. This reduced avidity in the anti-TNF–treated group was independent of the type of anti-TNF Ab used (infliximab or adalimumab; Supplemental Figure 9).

We next performed several sensitivity analyses to further interrogate Ab avidity. First, given that avidity maturation develops over time, we evaluated whether the time between vaccination and sampling impacted the avidity readouts. Within each timepoint analyzed, the time of sampling relative to vaccination was indistinguishable between study groups (Supplemental Figure 10A). Moreover, regression analyses indicated that the time between vaccination and sampling was not predictive of the avidity of anti-S IgG, likely reflecting the relatively homogeneous sampling times (Supplemental Figure 10B). We also assessed the impact of the time between anti-IL-12/23 or anti-TNF infusion and blood sampling on Ab avidity. For samples in which we had the patient self-reported date of most recent infusion, regression analyses revealed no impact of infusion timing on the avidity measurements (Supplemental Figure 11).

We also observed that the avidity of anti-S IgG measured here was positively associated with our previously reported neutralization capacity for ancestral SARS-CoV-2 after dose 2 and after dose 3 (Figure 7B). Thus, higher avidity IgG predicts greater neutralization capacity against the ancestral strain of virus.

Given that MBCs and LLPCs (a source of plasma IgG) are both cellular outputs of GCs, we examined whether the SHM analyses from BCR-Seq predicted plasma IgG avidity by linear regression analyses. Indeed, we observed that the frequency of V_H_ mutations in total S-specific MBCs (cMBCs, MZ-like B cells, and aMBCs pooled) or S-specific cMBCs alone predicts the avidity of plasma S-specific IgG (Figure 7C). Patients with IBD treated with anti-TNF had less SHM, and this predicted a lower avidity response (Figure 7C).

By analysis of the proportion of Abs that resist successive washes with ammonium thiocyanate, the responses can be binned into binding categories, ranging from very low–to–high avidity, for each study group (Figure 7D). After 1 dose of vaccine, we observed that the majority of antigen-specific IgGs were of very low or low avidity in all groups (Figure 7D). With successive doses of vaccine, the percentage of very low and low avidity IgG decreased, with a corresponding increase in the percentage of medium and high avidity IgG, indicative of affinity maturation (Figure 7D). Compared with healthy controls, after 2 and 3 vaccine doses, patients with IBD treated with anti-TNF had a significantly higher percentage of medium avidity S-specific IgG and a correspondingly lower percentage of high avidity IgG (Figure 7, D and E). Overall, these data demonstrate that vaccine-induced responses in patients treated with anti-TNF show reduced and delayed affinity maturation relative to the healthy control group or patients treated with anti-IL-12/23.

## Discussion

We and others have previously reported a decreased magnitude and persistence of Ab responses to SARS-CoV-2 mRNA vaccination in patients with IMID treated with anti-TNF (8–11). In our previous study, decreased S-specific Ab levels were observed only in patients with IBD treated with anti-TNF, whereas S-specific Ab responses of patients with rheumatic disease were not affected, potentially due to the lower doses of anti-TNF agents used (8). Here, we performed an in-depth investigation of the effect of anti-TNF on vaccine-induced GC outputs in patients with IBD. We observed reduced S-specific BCR SHM, as well as decreased and delayed affinity maturation in patients with IBD treated with anti-TNF compared to people in the healthy control group or patients treated with anti-IL-12/23. We also observed a decreased frequency of vaccine-induced cMBCs. As patients with IBD treated with anti-IL-12/23 were largely indistinguishable from people in the healthy control group in their Ab responses and MBC phenotypes yet have the same age distribution and disease state as the patients treated with anti-TNF, with the majority in remission at the time of the study, they serve as an ideal control to assess the effect of anti-TNF treatment, independent of any effects of disease. Together, these data support the conclusion that patients with undergoing treatment for IBD with anti-TNF have reduced and delayed vaccine-induced affinity maturation, consistent with impairment of the GC reaction.

Based on single-cell RNA-Seq, we found that a larger fraction of S-specific MBCs derived from patients with IBD treated with anti-TNF were aMBCs and MZ-like B cells, whereas cMBCs were the most frequent population in the healthy control group and patients treated with anti-IL-12/23. This is likely due to a decrease in cMBC output from the GC, rather than an expansion of extrafollicular responses based on our additional immunophenotyping analysis. Flow cytometric analysis of PBMC showed a reduction in the frequency of antigen-specific aMBCs and cMBCs relative to total populations of these subsets, whereas the S-reactive B cell frequencies among the MZ-like B cell subset did not change between groups.

In contrast with the impact of anti-TNF on vaccine-induced S-binding MBCs, immunophenotyping of total B cell subsets (DN, cMBCs, MZ-like B cells, and naive B cells) revealed no major differences in MBC subsets between the study groups. The impact of anti-TNF on a specific vaccine-induced response, but lack of detectable impact on overall MBC populations, may reflect that much of the MBC repertoire is established during childhood and fine tuned with subsequent exposures (52). Meanwhile, the peak ages of diagnosis for Crohn’s disease and ulcerative colitis are 20–30 years and 30–40 years, respectively (53), in line with the median age of diagnosis in our IBD cohort. This suggests that immune responses to vaccination prior to anti-TNF treatment may be intact, while responses to vaccinations delivered while on anti-TNF treatment are likely to be impaired in this group. This likely extends to other antigens, as impaired humoral responses in patients with IBD treated with anti-TNF have also been demonstrated in the context of influenza vaccination. Patients with IBD treated with infliximab (anti-TNF) had lower levels of Abs specific for influenza A/H3N2 or A/H1N1 compared with people in the healthy control group, whereas patients with IBD treated with ustekinumab (anti-IL-12/23) had similar responses to influenza vaccination as people in the healthy control group (17), similar to our findings with SARS-CoV-2 vaccines.

Although evidence from mouse knockout studies and limited human studies suggested that TNF blockade would impair the GC reaction (20, 21, 25), it was not predictable how substantially anti-TNF maintenance therapy, rather than complete knockout, would impact affinity maturation. Notably, we observed significantly less SHM in the V_H_ and V_L_ regions of S-specific MBCs in patients treated with anti-TNF, characterized by fewer replacement mutations in CDR1–3. Correspondingly, across 4 vaccine doses, S binding plasma IgG Abs from patients treated with anti-TNF were of lower overall avidity, with a lower proportion of high affinity Abs compared with patients treated with anti-IL-12/23 or people in the healthy control group. We also found that the level of SHM in MBC BCRs predicted both the avidity and neutralization activity of plasma Abs. While SHM measurements were derived from MBCs and avidity was assessed from LLPC-derived IgG, the positive association between these measurements likely reflects that both measurements are reading out the quality of the GC response. Together, these data support the conclusion that patients with undergoing treatment for IBD with anti-TNF have reduced and delayed vaccine-induced affinity maturation, consistent with impairment of the GC reaction.

A prior study of vaccinated nursing home residents and health care workers reported that the affinity of S-specific IgG Abs increased after the third dose of SARS-CoV-2 mRNA vaccine, with no further increase after the fourth dose (54). Similarly, our assessment of patients with IBD treated with anti-TNF and anti-IL-12/23 showed that S-specific Ab avidity plateaued after 3 vaccine doses, albeit the anti-TNF group did not reach the same level of avidity as the anti-IL-12/23–treated group.

Another study of SARS-CoV-2 vaccinated and treated patients with IBD reported a defect in the affinity maturation of the Ab response of patients with IBD after 2 doses of vaccine, based on decreased S1-RBD tetramer binding to IgG^+^ MBCs (55). The study reported that the defect in affinity maturation was corrected after a third dose of vaccine; however, the study did not segregate the after-third dose results of the IBD group by treatment type or compare the IBD group to the healthy control group that received a third dose. In contrast, our study showed that anti-S Abs from anti-TNF–treated participants were of lower overall avidity than Abs from patients treated with anti-IL-12/23, with a notably reduced proportion of high avidity Abs, even after 4 doses of vaccine. Our study used an established ELISA-based avidity assay (56) incorporating step-wise increases in ammonium thiocyanate concentration, enabling the detection of a broad range of Ab avidities, and this may have increased the sensitivity of our assay over a flow cytometry–based tetramer approach.

Although S-specific IgG responses were reduced in the anti-TNF group, S-specific IgM was not significantly different between groups. If CSR was substantially impaired, one would have expected increased IgM responses. CSR has been shown to take place largely before entry of B cells into the GC and before the onset of somatic hypermutation (57). Furthermore, in TNF knockout mice, CSR still occurs in the absence of primary B cell follicles or GC, although T-dependent IgG responses were impaired (20). Therefore, the apparent increase in the proportion of S-specific IgM^+^ MBCs in the anti-TNF group likely reflects the lower frequencies of IgG^+^ cMBCs due to decreased GC outputs, rather than an increase of IgM^+^ MBCs because of incomplete CSR.

We detected a range of frequencies of SHM in the BCRs of MBC, from unmutated to highly mutated clones, indicating that sorting S-PE tetramer binding MBCs before sequencing did not selectively enrich for only high affinity clones. Thus, despite the small number of memory B cells analyzed, we believe that we have analyzed a relatively representative repertoire. The diversity of S-specific MBCs did not appear to be reduced in patients with IBD treated with anti-cytokines compared with people in the healthy control group, thus, we did not observe selective expansion of specific, high avidity clonotypes. Polyclonal repertoires of high diversity are expected, given the stochastic nature of V, D, and J gene recombination during B cell development and the large number of clonotypes we detected from BCR-Seq. However, our study was limited by the small number of BCRs analyzed.

Analysis of BCR repertoire revealed high and similar diversity among healthy controls and patients with IBD who were treated; thus, anticytokine Abs do not appear to impact the repertoire of S-specific MBCs. We observed similar V_H_- and V_L_-biased gene usage across study groups as well as agreement with the CoV-AbDab database and published reports with respect to highly represented V_H_ genes for S or RBD specificities in convalescent or vaccinated individuals (58–61). We also observed public Ab responses in the light chains; *IGKV1-33* and *IGKV1-19* have been reported to be the most used among RBD-specific Abs, while *IGKV3-20* and *IGKV3-11* are the most used among S2 Abs (62). We detected 48 paired heavy and light chain variable genes shared between patients with IBD treated with anti-cytokines and people in the healthy control group and these pairs had been previously reported in a large-scale systematic survey of Ab responses to SARS-CoV-2 (62), consistent with antigen-driven selection. Thus, the overall biased and similar gene usage of S-specific BCRs across our study groups and other studies further supports the convergent evolution of SARS-CoV-2 vaccine–induced B cell responses.

Limitations of our study include the small number of individuals in each study group and the limited number of cells analyzed in the RNA-Seq data due to constraints on availability of matched patient samples across timepoints from the original IMPACT study and the low frequency of circulating MBCs. Although the anti-TNF- and anti-IL-12/23–treated IBD study groups were heterogenous in terms of type of infusion medication, dosage, and frequency of infusion, the heterogeneity in the type of medication was eliminated in our RNA-Seq analyses by assessing only patients treated with infliximab in the anti-TNF–treated group or patients treated with ustekinumab in the anti-IL-12/23–treated group. Moreover, in our assessments of plasma IgG avidity, we were powered to stratify patients with IBD treated with anti-TNF based on the type of Ab infusion medication (infliximab or adalimumab) and saw no differences between groups. Another limitation of our study is the focus on memory B cells derived from peripheral blood rather than cells derived from draining lymph nodes, precluding analysis of GC B cells from secondary lymphoid tissues (63). The effects of sampling location are expected to be minimal, however, as clonal overlap between circulating and secondary lymphoid populations has been reported after vaccination (64). As such, our interpretation on the GC reaction is limited to analysis of GC outputs rather than on GC structure.

In summary, our study presents an in-depth analysis of the humoral immune response to SARS-CoV-2 mRNA vaccination in patients with IBD who were treated with anti-TNF or anti-IL-12/23 compared with people in the healthy control group. Our findings suggest that maintenance treatment of patients with IBD with anti-TNF Abs, but not anti-IL-12/23, impairs and delays affinity maturation of a vaccine-induced Ab response, leading to reduced quantity and quality of GC outputs. These data suggest that patients on anti-TNF maintenance therapy may require additional vaccine boosters to compensate for a delayed and impaired affinity maturation response.

## Methods

### Sex as a biological variable

All study groups contained male and female participants. When powered to stratify by sex, we did not observe sex differences to be a significant predictor of anti-S Ab levels or avidity. Sex was controlled for in regression models.

### Study design

This study used residual stored samples from the IMPACT prospective observational cohort, which investigated immune responses to SARS-CoV-2 mRNA vaccination in a cohort of patients with IMIDs (8, 9). Plasma and PMBCs were isolated from healthy controls and patients with IMIDs at up to 8 timepoints spanning pre and post 1 to 4 doses of SARS-CoV-2 mRNA vaccine (BNT162b2 Pfizer-BioNTech or mRNA-1273 Moderna) (Figure 1A) (8, 9). Here, we used samples from patients with IBD treated with anti-IL-12/23 or anti-TNF biologics, and healthy controls. Participants were selected based on similar demographics (age, sex, BMI, disease state) and the availability of samples from the timepoints of interest. All participants were uninfected.

### Generation of S-streptavidin phycoerythrin tetramers

The expression, purification and biotinylation of the ancestral SARS-CoV-2 S ectodomain was performed as previously described (65). The full description of the construct is provided in the Supplemental Methods. Of note, the construct contained specific changes to stabilize the prefusion conformation. Streptavidin R-Phycoerythrin conjugate (SA-PE) (premium grade, Invitrogen, S21388) was used to make the S-SA-PE complexes. Biotinylated-S in PBS was mixed with SA-PE at molar ratio of 8:1 to 6:1 (S:SA-PE) for 2 hours at room temperature, and the SA-PE-S complex was purified by gel filtration (Superose 6 Increase column, Cytiva). Visualization of the complex by negative stain electron microscopy revealed the complex contained 2 trimeric S proteins per SA-PE complex. The complex was stored in PBS with protease inhibitor (Complete mini protease inhibitor-EDTA free, Roche-11836170001) at 4°C. The S-SA-PE tetramers are referred to as “S-PE” tetramers in the manuscript and figures.

### Single-cell RNA-seq

#### Sample preparation.

Samples taken at 3–4 months after vaccine dose 2 and 3 were used for scRNA-seq and included 3 people in the healthy control group, 5 patients with IBD treated with anti-TNF, and 4 patients with IBD treated with anti-IL-12/23 (Table 1) with matching between-time points for all but 1 subject. B cells were isolated from cryopreserved PBMCs (Militenyi Pan-B Cell Isolation Kit), blocked with streptavidin (Promega) and anti-human Fc receptor block (Invitrogen), then stained with 200 ng of S-PE tetramers for 40 minutes at 4°C. Cells were subsequently stained for 20 minutes at 4°C with an Ab cocktail and labeled with barcoded TotalSeq-C Hashtag Abs 1–12 (BioLegend) (Supplemental Table 6) to facilitate downstream demultiplexing of samples. S-PE tetramer-bound MBCs were sorted using a BD FACSymphony S6 SE analyzer (Figure 1, A and B).

#### Single-cell RNA-seq library preparation.

Sorted single cells were encapsulated in droplets using the Chromium GEM-X Single Cell 5’ Kit v3. 5’ Cellular Indexing of Transcriptomes and Epitopes by Sequencing (CITE-Seq) gene expression libraries (10X Genomics, Single Cell 5’ R2-only v3) and BCR V(D)J libraries (10X Genomics, Single Cell V(D)J R2-only v3) were prepared and sequenced on an Illmunia NovaSeq X Plus platform, with a target median sequencing depth of 50,000 read pairs per cell for gene expression libraries and 5,000 read pairs per cell for BCR and CITE-Seq libraries. Two datasets were generated, one for each timepoint: 3–4 months after dose 2 or after dose 3.

#### Single-cell gene expression processing and data analysis.

FASTQ paired-end reads were processed using 10x Genomics’ Cell Ranger (v.8.0.0.0; “count” command) and aligned against the human reference GRCh38-2024-A. The resulting filtered UMI count matrix generated from the cellranger pipeline was loaded to generate Seurat objects (66). Cells were excluded from analyses if the percentage of mitochondrial genes > 10%, number of unique features < 200, or the number of unique features > 6500. Gene counts were normalized using Seurat’s NormalizeData function. TotalSeq-C hashtags were normalized by centered log-ratio (CLR) normalization. Samples were demultiplexed using the HTODemux function and cell identities were established based on the max HTO signal. Cells having a margin of less than 2.0 between the max and secondary max HTO signal were excluded to prevent incorrect calling of the cell’s identity. Principle component analyses were performed using the top 2000 variable features (regressing out isotype genes). Data from both timepoints were integrated using Harmony (67) and dimensionality reduction was performed using UMAP.

#### Differential gene expression analysis.

Differentially expressed genes (DEGs) between clusters were detected using the Seurat FindAllMarkers function and considered differentially expressed if the adjusted P value < 0.05 and average log_2_(fold change) > 0.6. The FindMarkers function was used to compare the same cluster between 2 study groups and DEGs were visualized on a VolcanoPlot.

#### BCR repertoire analysis.

V(D)J contigs were processed using the Immcantation framework (v4.5.0) (68). Contigs were aligned to the IMGT reference using IgBLAST (v4.5.0) (68, 69). Contigs containing nonproductive sequences, missing a heavy chain or having multiple heavy chains, or lacking corresponding gene expression data were removed from analyses. Clonal analysis based on BCR heavy chain was conducted using SHazaM (68). The SCOPer hierarchialClones function was used to assign Ig sequences to clones, which utilizes a hierarchical clustering approach to define clones as groups of cells sharing common V and J gene annotations and CDR3 length (68, 70). Germline sequences were generated for each clone using Dowser and the IMGT reference database of known alleles (71, 72). SHazaM was used to calculate BCR SHM frequencies. Alakazam was used to perform diversity analyses and analyze Ig gene usage (68). Gene usage frequencies were compared with the CoV-AbDab public database of Abs and nanobodies able to bind coronaviruses (46), which we filtered to only include entries reporting human Ig genes and having specificity for SARS-CoV-2.

### Flow cytometry

Cyro-preserved PBMCs were plated at 5 million cells per well, blocked with streptavidin and anti-human Fc receptor block (Invitrogen), then stained with 200 ng of ancestral SARS-CoV-2 S-PE tetramer per well for 40 minutes at 4°C. Cells were subsequently stained for 20 minutes at 4°C with a surface Ab cocktail (Supplemental Table 7) and acquired on a BD FACSymphony A3 cell analyzer.

### ELISA

Plasma samples were diluted from 1:5 to 1:327,680 in 1% Blocker BLOTTO in PBST, for a total of 10 dilutions per sample. IgG and IgM Ab responses against the full-length ancestral SARS-CoV-2 S trimer were measured using a chemiluminescent ELISA assay as previously described (73). Effective concentration (EC_50_) values were calculated using the nlsLM function (74) and represent the plasma concentrations that give a response halfway between the assay’s minimum value (blank controls) and a sample’s maximum value. Details on data normalization and analysis are provided in the Supplemental Materials in Supplemental Methods.

### Anti-S IgG avidity assay

High-binding plates (Sarstedt) were coated overnight at 4°C with 1 μg/ml of trimeric ancestral SARS-CoV-2 S protein, prepared as previously described (65). A positive control consisting of a pooled plasma mixture from 8 SARS-CoV-2 mRNA vaccinated (34 or more doses) healthy individuals, with or without natural infection, was used throughout. Plasma was heat-inactivated for 30 minutes at 56°C. Antigen-coated plates were blocked with blocking buffer (1 X TBS + 0.1% Tween 20 + 2% BSA) at room temperature (RT). Patient plasma was serially diluted (3-fold, 7-point serial dilution from 1:100) with dilution buffer (1 X TBS + 0.1% Tween 20 + 0.1% BSA) and 100 μl was added to blocked plates in triplicates and incubated at RT for 1 hour. To assess avidity, 200 μl of dilution buffer, 0.5 M ammonium thiocyanate (NH_4_SCN), 1 M NH_4_SCN, or 2 M NH_4_SCN were added to appropriate wells, with a 15 minute incubation at RT. 100 μl of horseradish peroxidase conjugated anti-human IgG, Fcγ specific (1:150,000 working dilution; Jackson ImmunoResearch) was then added to each well and incubated for 1 hour at RT. 100 μl of HRP substrate (1-Step Ultra TMB-ELISA substrate solution; ThermoFisher) was added. After 10 minutes of development, 50 μl of 2 M sulfuric acid was added and optical densities were measured at 450 nm. Avidity is reported as relative avidity index (%), fractional relative avidity index (%), and total relative avidity index (AU; avidity units). Calculations of avidity (Supplemental Table 5) were adapted from a previously reported assay (56).

### Statistics

Longitudinal multivariate regression analyses (STATA 18) of IgM and IgG levels controlled for age, sex, BMI, and vaccine type, with an interaction term between timepoint and study group. The aforementioned factors, with an additional term for IgG Ab concentration, were controlled for in analyses of IgG avidity. For nonlongitudinal comparisons between study groups, where applicable, linear regressions (STATA 18) controlled for age, sex, BMI, and vaccine type or Kruskal-Wallis 1-way ANOVA with Dunn’s multiple comparisons tests (GraphPad Prism) were performed. Wilcoxon matched pairs signed rank 2-tailed tests (GraphPad Prism) were performed to compare paired measurements across 2 timepoints. In all analyses, a *P* value of less than 0.05 was considered significant. Data represent mean ± 95% CI, unless otherwise noted.

### Study approval

This study was approved by the ethics boards of the University of Toronto (research ethics board [REB] protocol 27673), Mount Sinai Hospital/Sinai Health System (MSH REB 21-0022-E), and University Health Network–Toronto Western Hospital division (REB 21-5096). Written informed consent was obtained from all participants prior to participation.

### Data availability

Raw and processed CITE-Seq and BCR-Seq data can be found in NCBI GEO, under accession number GSE290006. Code used in this study are available in GitHub (https://github.com/michelle-cheung25/Cheung_JCI2025; commit ID 4e55912). Values for all data points in graphs are reported in the Supporting Data Values file.

## Author contributions

MWC, SX, FQ, MDB, KC, RMD, and THW designed or conducted experiments, acquired and/or analyzed data. MWC prepared figures; MWC and THW drafted the manuscript. JRS assisted with the recruitment of and processing of healthy donor blood for assay optimization, sample prep for single-cell RNA-Seq and provided guidance with statistical analyses. YL and JMR generated the ancestral S proteins and S-PE tetramers for the following experiments: single-cell RNA-Seq, flow cytometry, avidity assays. JDL and JMS assisted with organizing and verifying patient metadata. VC, MSS, ACG, and THW designed the IMPACT study and obtained research funding for the study. All authors edited and reviewed the manuscript.

## Supplementary Material

Supplemental data

ICMJE disclosure forms

Supporting data values

## Figures and Tables

**Table 1:**
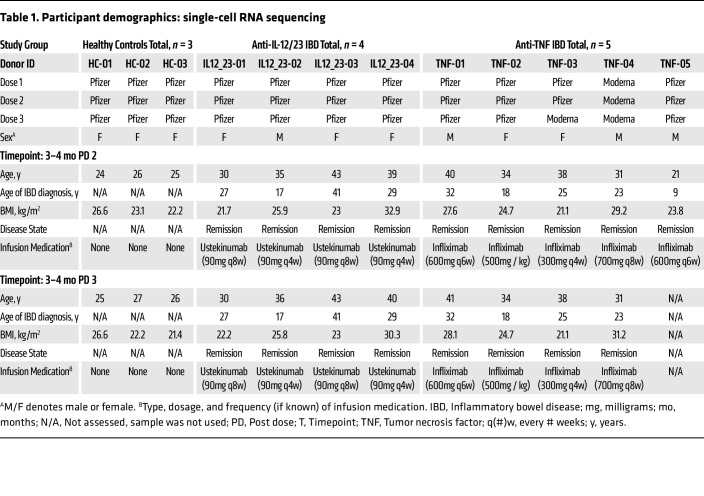
Participant demographics: single-cell RNA sequencing

## Version 1 07/29/2025

In-Press Preview

## Version 2 10/01/2025

Electronic publication
