# Supplementary material for: Reduced vaccine-induced germinal center outputs in patients with inflammatory bowel disease treated with anti-TNF biologics
Source: J Clin Invest. 2025 Jul 29;135(19):e192589. doi: 10.1172/JCI192589 (PMC12483611; doi:10.1172/JCI192589)
Supplement: Supplemental data [file jci-135-192589-s262.pdf]

Supplemental Material

Supplemental Methods: Page 2-3

Supplemental Figures: Page 4-19

Supplemental Tables: Page 20-27

Corresponding manuscript:

Reduced vaccine-induced germinal center outputs in inflammatory bowel disease patients treated with anti-TNF biologics

Authors: Michelle W. Cheung,[1] Samantha Xu,[1] Janna R. Shapiro,[1] Freda Qi,[2] Melanie Delgado-Brand,[2] Karen Colwill,[2] Roya M. Dayam,[2] Ying Liu,[3] Jenny D. Lee,[2,4] Joanne M. Stempak,[2,4] James M. Rini,[3,5] Vinod Chandran,[6,7,8,9] Mark S. Silverberg,[2,4,10] Anne-Claude Gingras,[2,3] Tania H. Watts,[1,*]

Affiliations:
[1]Department of Immunology, University of Toronto; Toronto, Ontario, Canada.
[2]Lunenfeld-Tanenbaum Research Institute at Mount Sinai Hospital, Sinai Health System; Toronto, Ontario, Canada.
[3]Department of Molecular Genetics, University of Toronto; Toronto, Ontario, Canada.
[4]Zane Cohen Centre for Digestive Diseases, Division of Gastroenterology, Mount Sinai Hospital, Sinai Health System; Toronto, Ontario, Canada.
[5]Department of Biochemistry, University of Toronto; Toronto, Ontario, Canada
[6]Gladman Krembil Psoriatic Arthritis Research Program, Schroeder Arthritis Institute, Krembil Research Institute, University Health Network; Toronto, Ontario, Canada
[7]Division of Rheumatology, Department of Medicine, University of Toronto; Toronto, Ontario, Canada
[8]Department of Laboratory Medicine and Pathobiology, University of Toronto; Toronto, Ontario, Canada
[9]Institute of Medical Science, University of Toronto; Toronto, Ontario, Canada
[10]Division of Gastroenterology, Department of Medicine, University of Toronto; Toronto, Ontario, Canada.

*Corresponding author: Tania H. Watts
Address: 1 King's College Circle, Toronto, Ontario, Canada
Telephone: 416-978-4551
Email: tania.watts@utoronto.ca

Supplemental Methods

Expression and purification of Wuhan SARS-CoV-2 Spike

The expression and purification of the Wuhan SARS-CoV-2 S ectodomain was performed as previously described (Abe KT, et al. A simple protein-based surrogate neutralization assay for SARS-CoV-2. *JCI Insight.* 2020;5(19)). Briefly, the soluble trimeric S protein ectodomain (residues 1–1211) was secreted from HEK293F cells with a C-terminal phage foldon trimerization motif followed by a 6 × His-tag and an Avi-Tag. S residues 682–685 (RRAR) were mutated to SSAS to remove the furin cleavage site and residues F817, A892, A899, A942, K986 and V987 were each mutated to proline to stabilize the prefusion conformation.

SARS-CoV-2 IgG and IgM ELISA data analysis

IgG and IgM Ab responses against the full-length Wuhan SARS-CoV-2 S trimer were measured using a chemiluminescent ELISA assay as previously described (Colwill K, et al. A scalable serology solution for profiling humoral immune responses to SARS-CoV-2 infection and vaccination. *Clin Transl Immunology.* 2022;11(3):e1380). Raw luminescence values from samples were normalized using the maximum and minimum luminescence values from control wells on the same plate. Maximum values were obtained from standard curves of recombinant Abs VHH72-Fc (National Research Council of Canada) and human anti-Spike S1 IgM (clone hIgM2001, GenScript, #A02046), for IgG and IgM isotypes, respectively. The minimum values were obtained from blank controls. A sigmoidal curve, with the association Y= Bottom + (Top-Bottom)/(1+10^(LogEC50-x)), was fitted to the results of each sample. Bottom values were constrained to 0. Effective concentration (EC50) values were calculated using the nlsLM function from the minpack.lm package in R and represent the plasma concentrations that give a

response halfway between the assay’s minimum value (blank controls) and a sample’s maximum value (top).

Supplemental Figures

**Supplemental Figure 1**. Detection of Wuhan SARS-CoV-2 S-specific memory B cells with S-Streptavidin Phycoerythrin (S-PE) tetramers.	**Page 5**
**Supplemental Figure 2**. Single-cell RNA-sequencing of S-specific memory B cells from each participant.	**Page 6**
**Supplemental Figure 3.** Transcriptomic analysis of S-specific memory B cells.	**Page 7**
**Supplemental Figure 4.** Differentially expressed genes in S-specific memory B cell subsets between study groups.	**Page 8-9**
**Supplemental Figure 5.** Immunophenotyping of peripheral blood B cell subsets.	**Page 10-11**
**Supplemental Figure 6**. Paired BCR heavy and light chain variable genes of S-specific memory B cells.	**Page 12**
**Supplemental Figure 7**. Paired BCR heavy and light chain variable genes of S-specific memory B cells shared by healthy controls and treated IBD patients.	**Page 13**
**Supplemental Figure 8.** Somatic hypermutation analyses of S-specific memory B cells.	**Page 14-15**
**Supplemental Figure 9.** Avidity of S-specific IgG Abs stratified by anti-TNF infusion medication.	**Page 16**
**Supplemental Figure 10.** Time since vaccination does not impact the avidity of S-specific IgG Abs over the time frames analyzed.	**Page 17-18**
**Supplemental Figure 11.** Timing of infusion medication relative to readouts does not affect the avidity of S-specific IgG Abs over the time frame analyzed.	**Page 19**

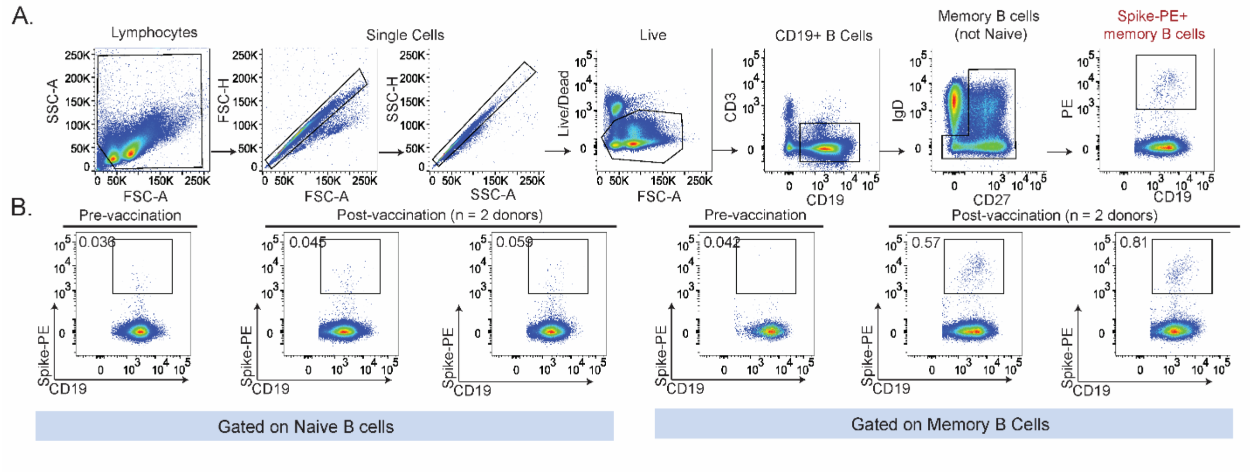

Supplemental Figure 1. Detection of Wuhan SARS-CoV-2 S-specific memory B cells with S-Streptavidin Phycoerythrin (S-PE) tetramers. (A) Gating strategy: lymphocytes > single cells > live cells > CD19^{+} B cells > memory B cells (NOT naïve B cells, which are IgD^{+}CD27^{-}) > Spike-PE tetramer bound memory B cells. (**B)** Flow plots from a pre-vaccination (n=1) donor and two post-vaccination (n=2) donors, gated on naïve B cells or memory B cells. Post-vaccination donors received three doses of vaccine. The pre-vaccination donor and post-vaccination donors are different individuals. The frequency of Spike-PE^{+} B cells is depicted in the upper left-hand corner of each plot.

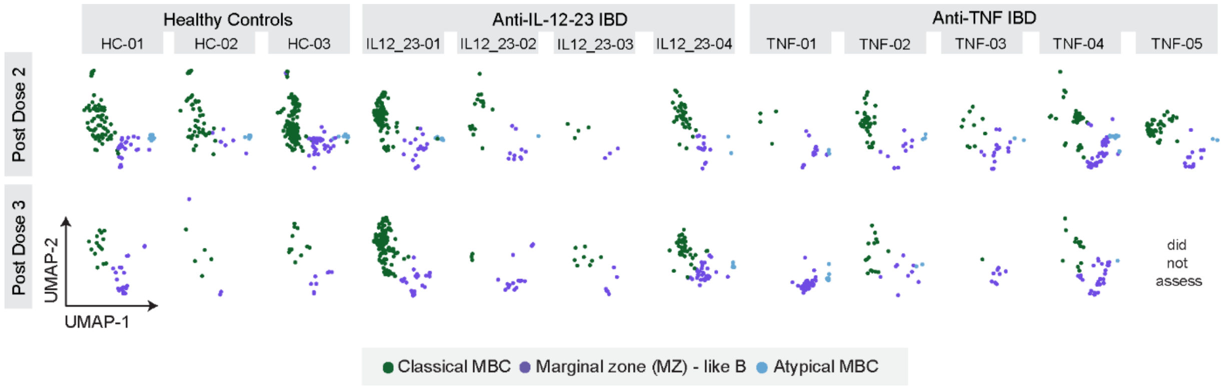

Supplemental Figure 2. Single-cell RNA-sequencing of S-specific memory B cells from each participant. Uniform manifold approximation and projection (UMAP) of S-specific memory B cells (MBC) per individual, colored by subtype and grouped by timepoint. Number of cells analyzed (by dose and patient identifier): post dose (PD) 2 HC-01 n = 107; PD3 HC-01 n = 36; PD2 HC-02 n = 71; PD3 HC-02 n = 8; PD2 HC-03 n = 179; PD3 HC-03 n = 19; PD2 IL12_23-01 n = 119; PD3 IL12_23-01 n = 142; PD2 IL12_23-02 n = 32; PD3 IL12_23-02 n = 23; PD2 IL12_23-03 n = 7; PD3 IL12_23-03 n = 14; PD2 IL12_23-04 n = 57; PD3 IL12_23-04 n = 77; PD2 TNF-01 n = 20; PD3 TNF-01 n = 45; PD2 TNF-02 n = 49; PD3 TNF-02 n = 26; PD2 TNF-03 n = 28; PD3 TNF-03 n = 9; PD2 TNF-04 n = 89; PD3 TNF-04 n = 45; PD2 TNF-05 n = 45; PD3 TNF-05 n = 66. Anti-IL-12/23 IBD: anti-IL-12/23 treated IBD patients; anti-TNF IBD: anti-TNF treated IBD patients.

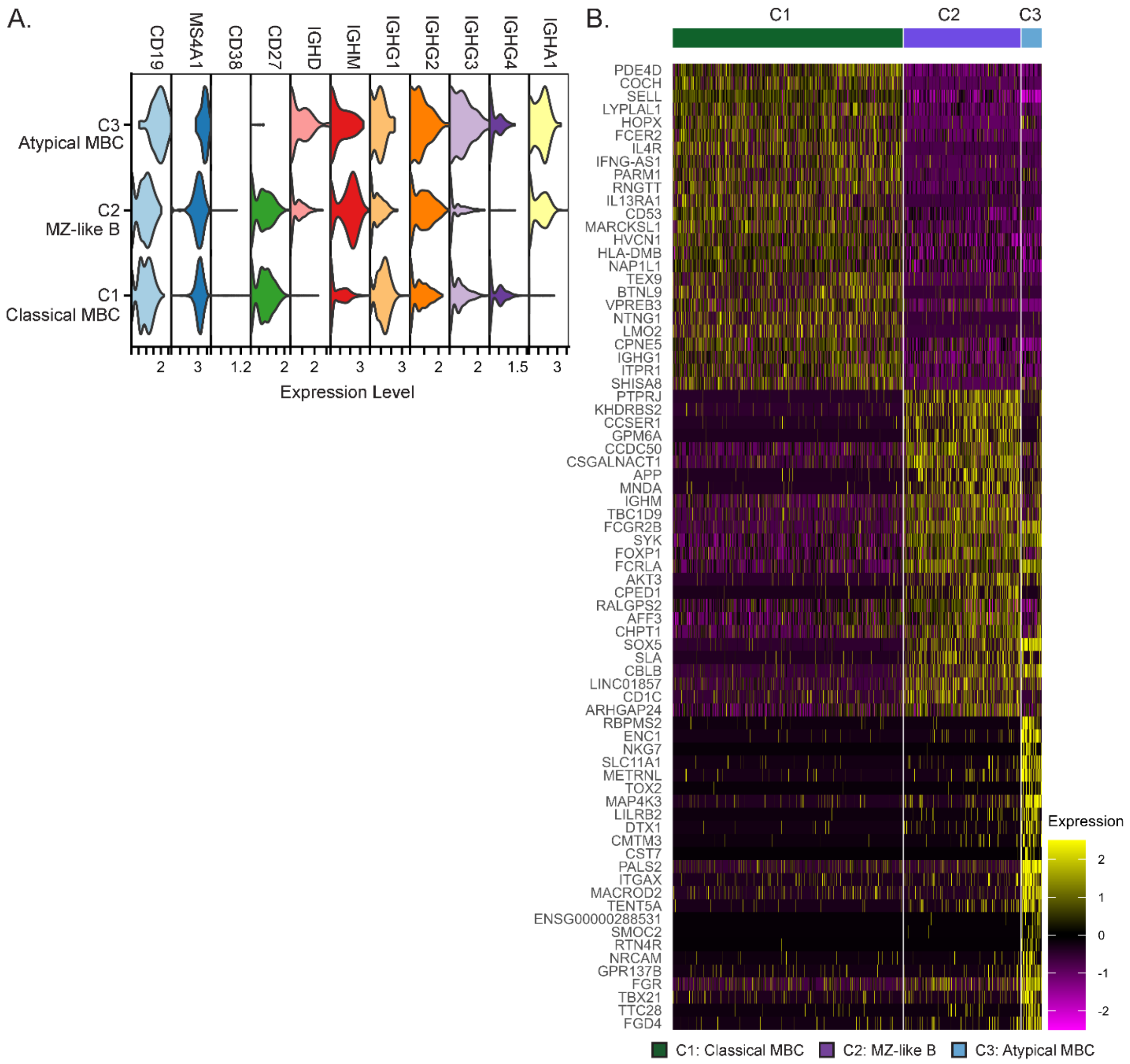

Supplemental Figure 3. Transcriptomic analysis of S-specific memory B cells. **(A)** Violin plots depicting the expression level of transcripts encoding surface markers in each subset of memory B cell. **(B)** Top 25 differentially expressed genes per memory B cell (MBC) subset (adjusted $P < 0.05$ and $\log_2$(fold change) > 0.6 or < -0.6). **(A-B)** Cluster 1 (C1): classical MBCs, Cluster 2 (C2): marginal zone (MZ) – like B cells, Cluster 3 (C3): atypical MBCs.

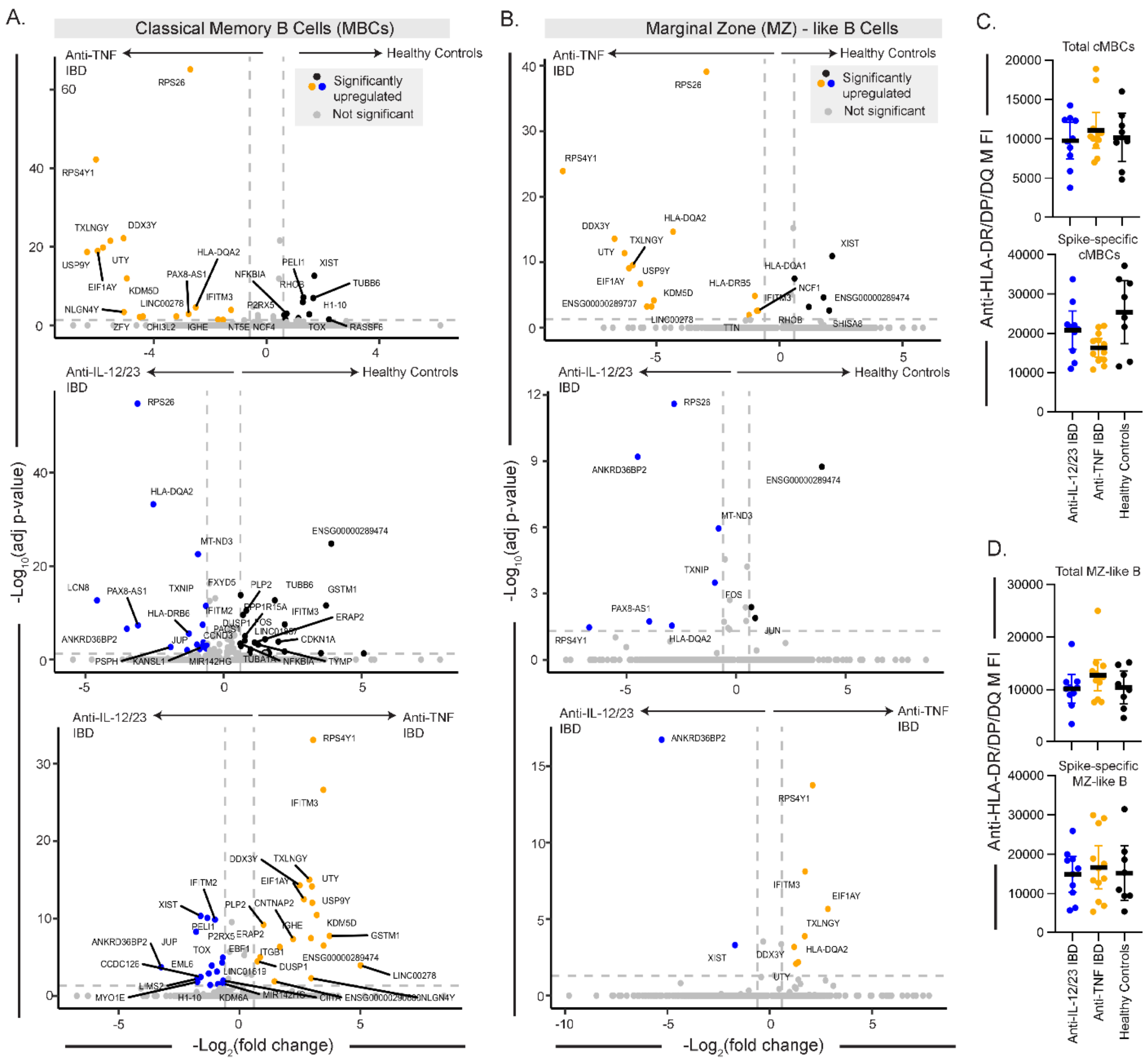

Supplemental Figure 4. Differentially expressed genes in S-specific memory B cell subsets between study groups. (A) Differentially expressed genes in S-specific classical memory B cells (MBC) (cluster 1; C1) from single-cell RNA-Seq. **(B)** Differentially expressed genes in S-specific marginal zone (MZ)-like B cells (cluster 2; C2) from single-cell RNA-Seq. **(A-B)** Comparisons between anti-TNF treated IBD patients vs healthy controls, anti-IL-12/23 treated IBD patients vs healthy controls, and anti-IL-12/23 treated IBD patients vs anti-TNF IBD treated IBD patients are shown. Significantly upregulated genes are colored in black (healthy controls),

orange (anti-TNF IBD) or blue (anti-IL-12/23 IBD) and considered significant if adjusted $P < 0.05$ (marked by the horizontal dashed grey line) and absolute value of average $\log_2$(fold change) > 0.6 (marked by the two vertical dashed grey lines). The top 50 significant genes are labeled. Non-significant genes are colored grey. S-specific classical MBCs and MZ-like B cells were pooled from post dose 2 and post dose 3. **(C-D)** Median fluorescence intensity (MFI) of anti-human HLA (pan HLA-DR/DP/DQ) in total and S-specific classical MBCs and MZ-like B cells, post dose 2, by study group. Assessed via flow cytometry. Data represent mean ± 95% CI. Kruskal-Wallis one-way ANOVA with Dunn's multiple comparisons tests were performed to compare frequencies between study groups; no significant differences were observed. Sample size: refer to Supplemental Table 1.

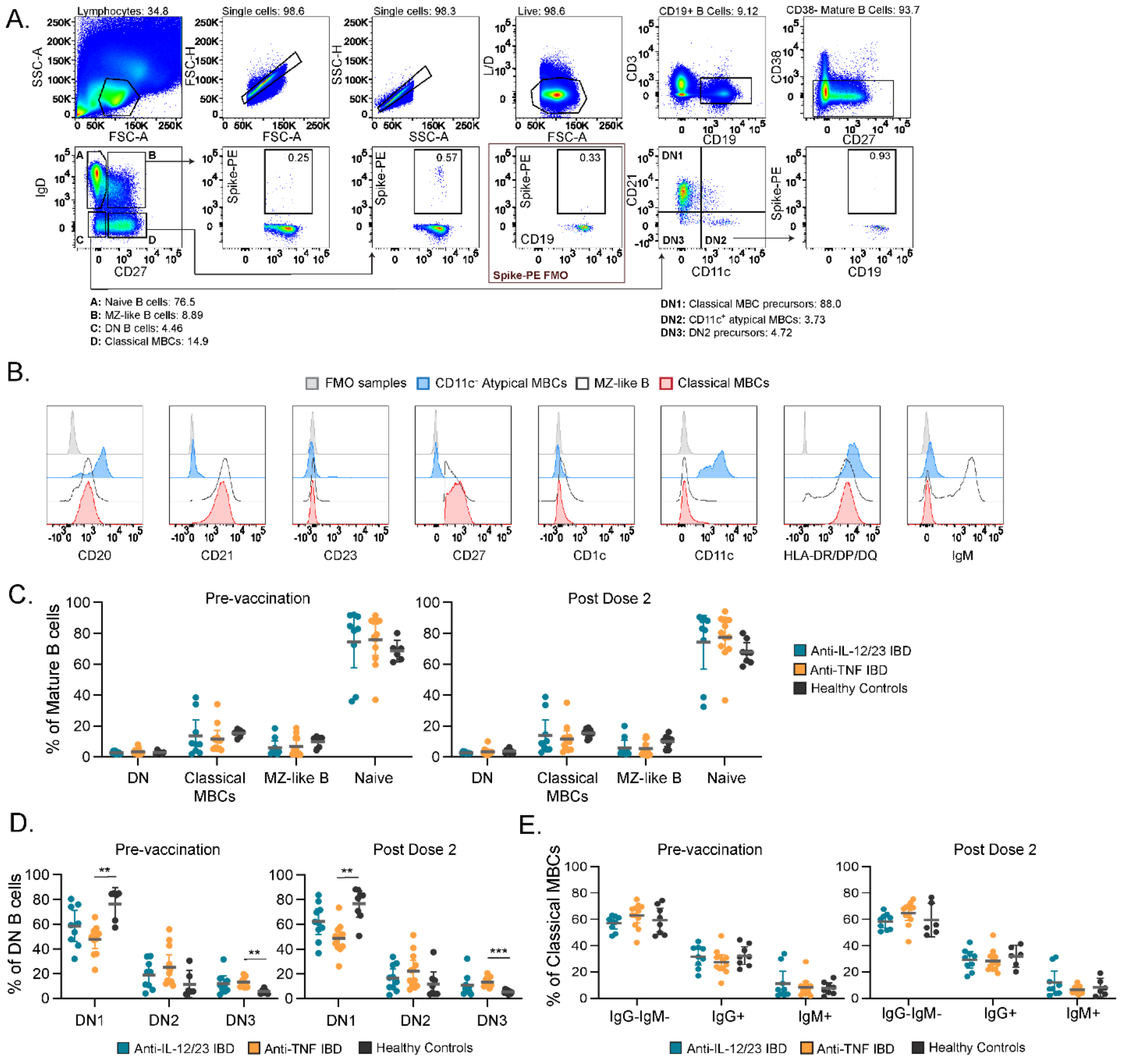

Supplemental Figure 5. Immunophenotyping of peripheral blood B cell subsets. (A) Representative gating strategy to detect total B cell subsets and Wuhan S-specific memory B cell (MBC) subsets. Gated on lymphocytes > single cells > live > CD3$^-$CD19$^+$ B cells > CD38$^-$ mature B cells > IgD vs CD27 > naïve B cells: IgD$^-$CD27$^+$; marginal zone (MZ) - like B cells: IgD$^+$CD27$^+$; double negative (DN) B cells: IgD$^-$CD27$^-$; classical MBCs: IgD$^-$CD27$^+$. From populations B and D > gate for S-PE tetramer positive MZ-like B cells and classical MBCs,

respectively. From population C > gate by CD21 vs CD11c to define DN B cell subsets: DN1: $CD21^{+}CD11c^{-}$; DN2 ($CD11c^{+}$ atypical MBCs): $CD21^{-}CD11c^{+}$; DN3: $CD21^{-}CD11c^{-}$. S-specific tetramer positive DN2 atypical MBCs were then gated for. **(B)** Representative histograms of the expression of selected markers in FMO samples, $CD11c^{+}$ atypical MBCs, MZ-like B cells, and classical MBCs. **(C)** Frequencies of total peripheral blood DN B cells, classical MBCs, MZ-like B cells, and naïve B cells as a percentage of $CD19^{+}CD38^{-}$ mature B cells, at pre-vaccination and 3-4 months post dose 2, by study group. **(D)** Frequencies of total peripheral blood DN B cell subsets (DN1, DN2, DN3) at pre-vaccination and 3-4 months post dose 2, by study group. **(E)** Ig isotype of total peripheral blood classical MBCs ($IgD^{-}CD27^{+}$). **(C-E)** Anti-IL-12/23 IBD: anti-IL-12/23 treated IBD patients; anti-TNF IBD: anti-TNF treated IBD patients. Anti-IL-12/23 treated IBD patients are colored in teal, anti-TNF treated IBD patients are colored orange, and healthy controls are colored black. For each subset of B cells, Kruskal-Wallis one-way ANOVA with Dunn's multiple comparisons tests were performed to compare frequencies between study groups. Data represent mean ± 95% CI. *$P < 0.05$, **$P < 0.01$, ***$P < 0.001$, ****$P < 0.0001$. Sample sizes are listed in Supplemental Table 1.

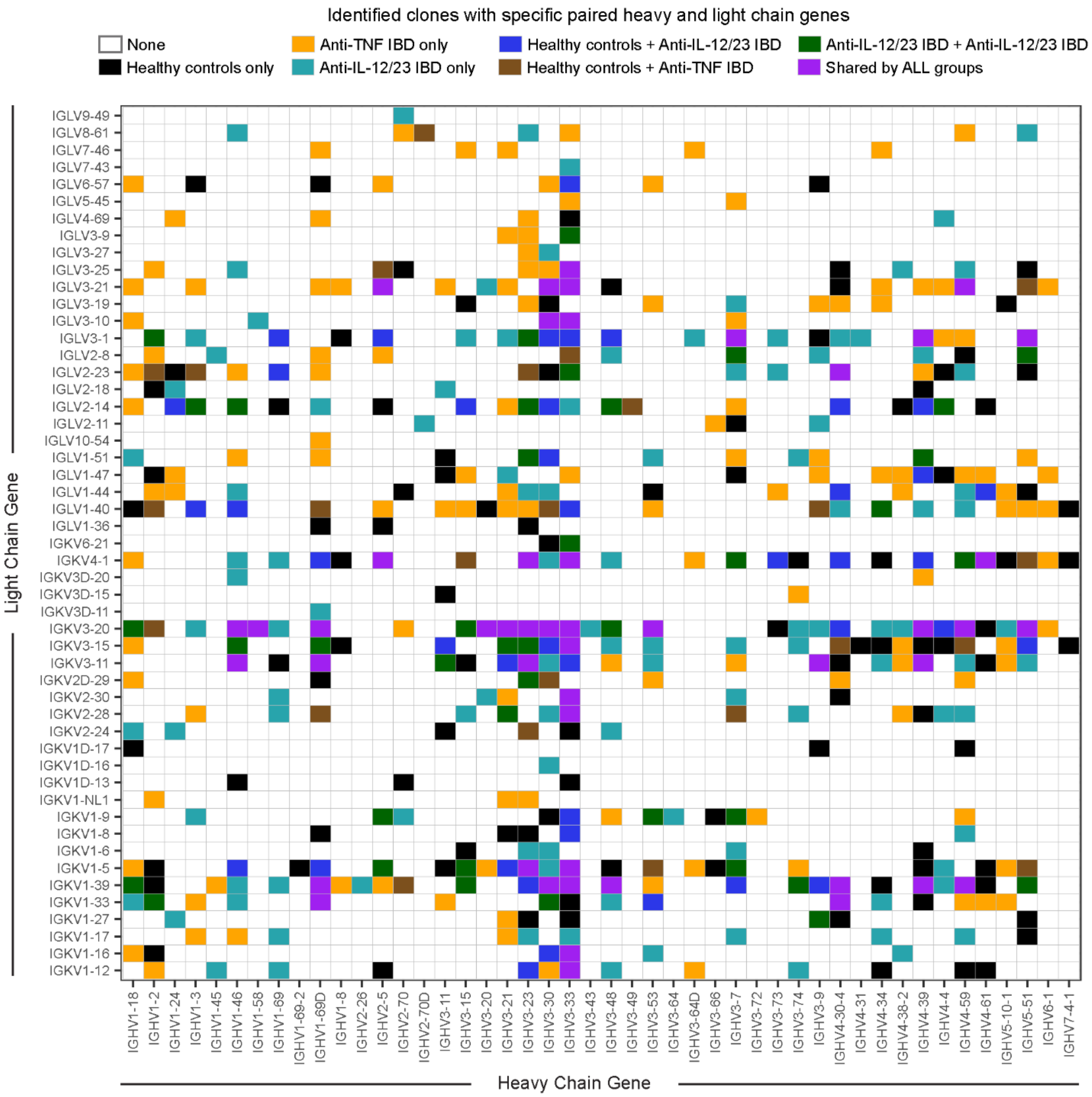

Supplemental Figure 6. Paired BCR heavy and light chain variable genes of S-specific memory B cells. Color coding represents in which combination(s) of study group(s) (healthy controls, anti-TNF treated IBD patients, anti-IL-12/23 treated IBD patients) a specific paired heavy and light chain gene was identified in. Purple colored squares represent gene pairs shared by all three study groups. Anti-IL-12/23 IBD: anti-IL-12/23 treated IBD patients; anti-TNF IBD: anti-TNF treated IBD patients.

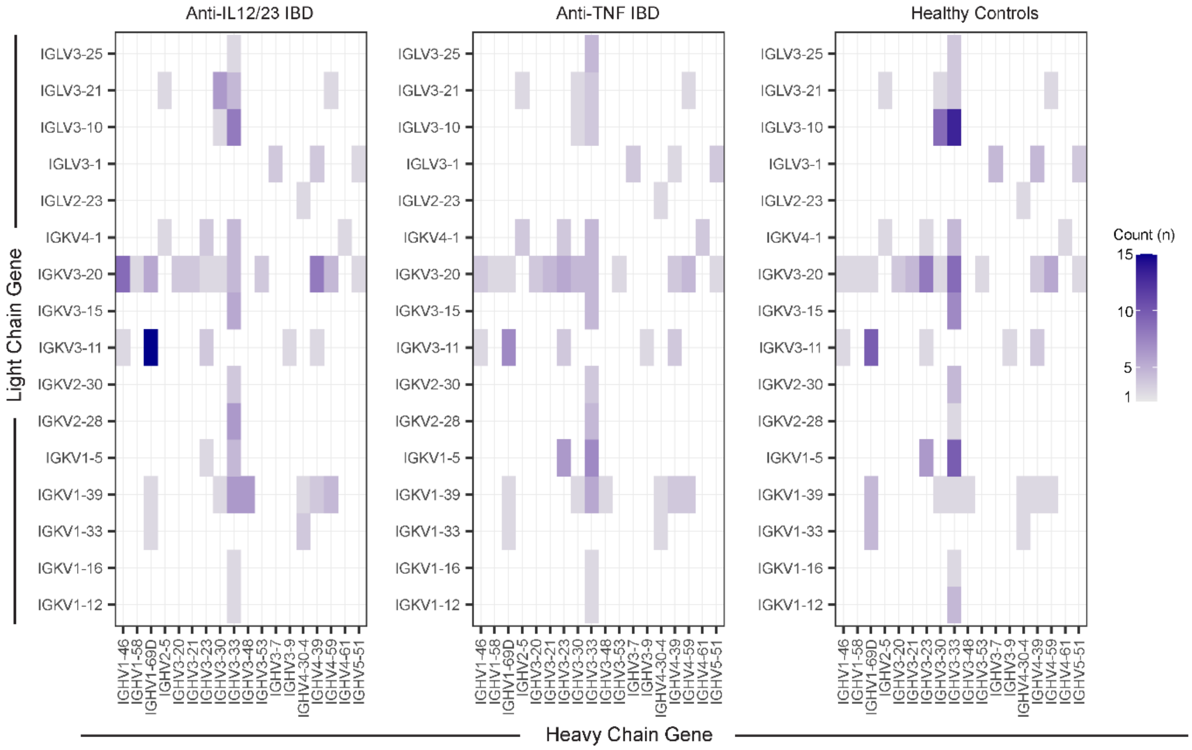

Supplemental Figure 7. Paired BCR heavy and light chain variable genes of S-specific memory B cells shared by healthy controls and treated IBD patients. 48 paired heavy and light chain variable genes shared by all three study groups (from Supplemental Figure 6). The heat maps depict the count of cells (n = # cells) expressing a specific heavy and light chain variable gene pair in each study group. Anti-IL-12/23 IBD: anti-IL-12/23 treated IBD patients; anti-TNF IBD: anti-TNF treated IBD patients.

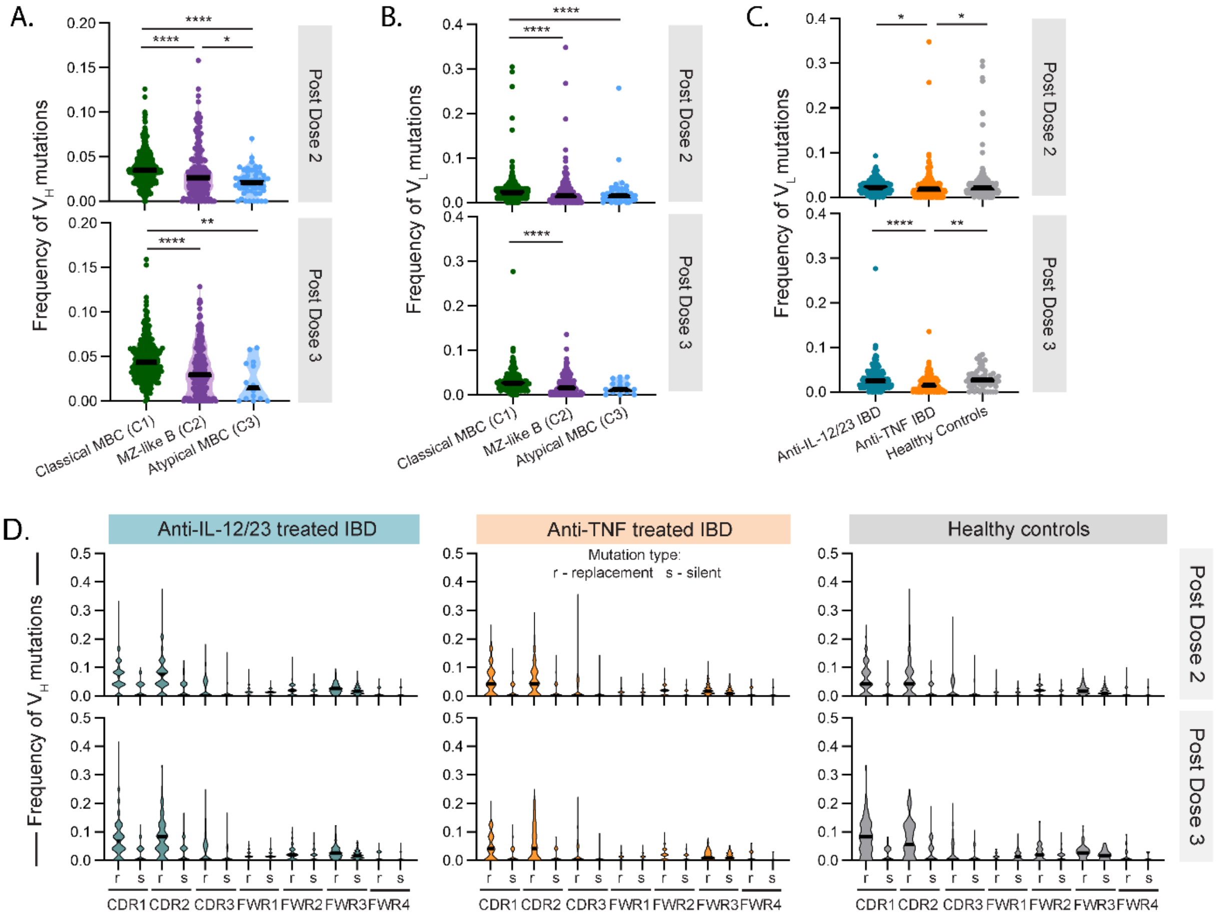

Supplemental Figure 8. Somatic hypermutation analyses of S-specific memory B cells. BCR-sequencing of S-specific memory B cells (MBCs); each dot represents a cell. Frequency of somatic hypermutations in the heavy chain **(A)** and light chain **(B)** variable region of S-specific MBCs (classical MBCs, marginal zone (MZ)-like B cells, and atypical MBCs pooled), post dose 2 and post dose 3, study groups pooled. **(C)** Frequency of somatic hypermutations in the light chain variable region of S-specific MBCs (classical MBCs, marginal zone (MZ)-like B cells, and atypical MBCs pooled), post dose 2 and post dose 3, grouped by study group. **(D)** Frequency of replacement and silent mutations in the complementarity determining regions (CDR) 1-3 and framework regions (FWR) 1-4 of the heavy chain variable regions of S-specific MBCs, post dose

2 and dose 3, grouped by study group. **(A-D)** Frequency is calculated as counts of mutations over the total number of positions in the V gene sequence or region (CDR/FWR). **(A-C)** Kruskal-Wallis one-way ANOVA with Dunn's multiple comparisons tests. *P < 0.05, **P < 0.01, ***P < 0.001, ****P < 0.0001. **(C-D)** Anti-IL-12/23 IBD: anti-IL-12/23 treated IBD patients; anti-TNF IBD: anti-TNF treated IBD patients.

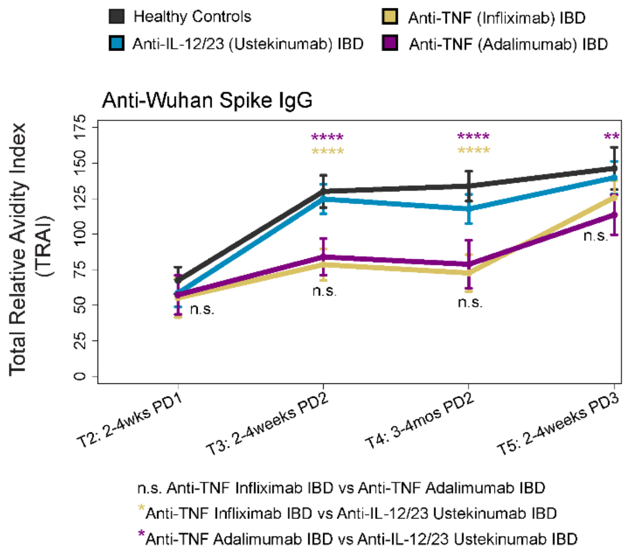

Supplemental Figure 9. Avidity of S-specific IgG Abs stratified by anti-TNF infusion medication. Avidity is reported as a total relative avidity index (TRAI). Longitudinal analyses of the avidity of Wuhan anti-S IgG in healthy controls (black), ustekinumab (anti-IL-12/23) treated IBD patients (teal), infliximab (anti-TNF) treated IBD patients (yellow), and adalimumab (anti-TNF) treated IBD patients (purple) across one to three doses of vaccine. Multivariate regression models controlled for age, BMI, sex, vaccine type, and IgG concentration, with an interaction term between time point (T) and study group. Asterisks in yellow indicate comparisons between infliximab treated IBD patients and anti-IL-12/23 treated IBD patients; asterisks in purple indicate comparisons between adalimumab treated IBD patients and anti-IL-12/23 treated IBD patients. *P < 0.05, **P < 0.01, ***P < 0.001, ****P < 0.0001. Non-significant (n.s.) p-values were derived from comparisons of infliximab treated IBD patients and adalimumab treated IBD patients at all timepoints. Samples sizes: refer to Supplemental Table 3.

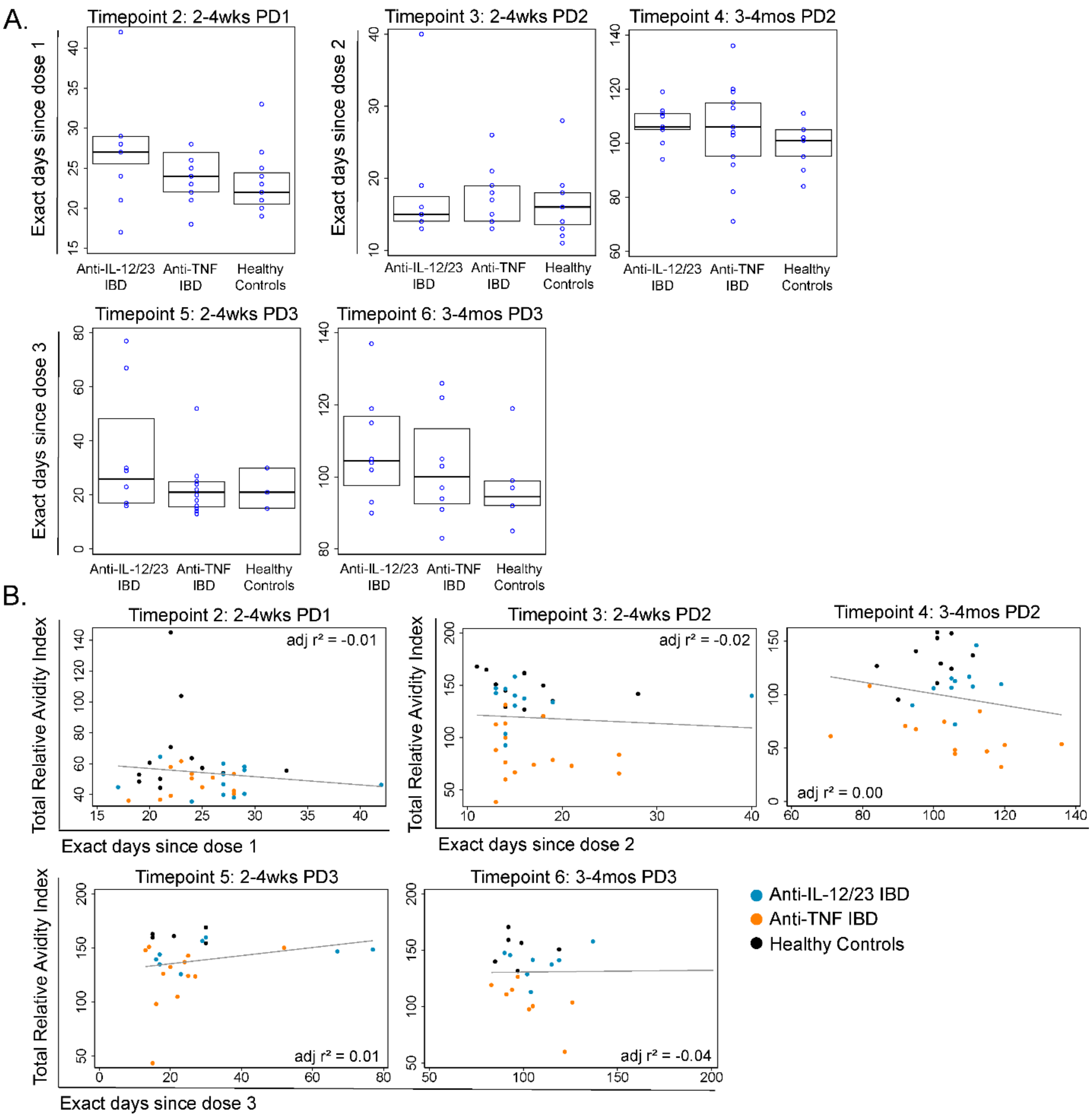

Supplemental Figure 10. Time since vaccination does not impact the avidity of S-specific IgG Abs over the time frames analyzed. (A) For each timepoint bin, the exact days since receiving vaccine doses 1, 2, or 3 are plotted for each study group. No significant differences between groups were observed. **(B)** Avidity is reported as total relative avidity index (TRAI). Within each timepoint bin, time (exact days) post vaccination (dose 1, 2, or 3) was plotted

against the avidity of Wuhan anti-S IgG. The grey lines depict linear regression modelling and adjusted r-squared values derived from modelling are listed for each timepoint. Teal circles represent anti-IL-12/23 treated IBD patients, orange circles represent anti-TNF treated IBD patients and black circles represent healthy controls. **(A-B)** Mos: months; PD: post dose; Wks: weeks

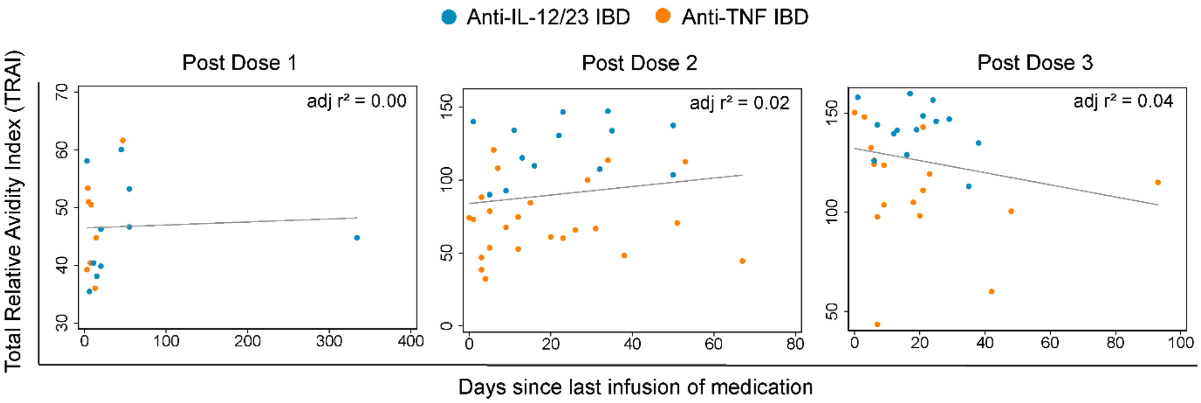

Supplemental Figure 11. Timing of infusion medication relative to blood sampling does not affect the avidity of S-specific IgG Abs over the time frame analyzed. Avidity is reported as a total relative avidity index (TRAI). Days since last infusion of medication (anti-IL-12/23 or anti-TNF) does not predict the avidity of Wuhan anti-S IgG, post 1-3 vaccine doses. Teal circles represent anti-IL-12/23 treated IBD patients and orange circles represent anti-TNF treated IBD patients**.** The grey lines depict linear regression modelling and adjusted r-squared values derived from modelling are listed.

Supplemental Tables

Supplemental Table 1. Patient demographics: immunophenotyping via flow cytometry. **Page 21**

Supplemental Table 2. Patient demographics: anti-Wuhan Spike IgG and IgM levels. **Page 22**

Supplemental Table 3. Patient demographics: anti-Wuhan Spike IgG avidity measurement. **Page 23**

Supplemental Table 4. Anti-Wuhan Spike IgG avidity measurement: comparisons between timepoint. **Page 24**

Supplemental Table 5. Calculation of relative avidity index, fractional relative avidity index and total relative avidity index. **Page 25**

Supplemental Table 6. Flow cytometry panel used to sort cells for single-cell RNA-Seq. **Page 26**

Supplemental Table 7. Flow cytometry panel to detect SARS-CoV-2 Spike-specific memory B cells. **Page 27**

Supplemental Table 1. Patient demographics: immunophenotyping via flow cytometry

Timepoint	T1: Pre-vaccination	T4: 3-4 MoPD2
**Healthy controls**		
Total, n	6	8
Female, n (%)	0 (0)	1 (12)
Age, y, median (IQR)	41 (34 - 47)	35.5 (27 - 47)
BMI, kg/m^2, median (IQR)	25 (24 - 27)	24 (23 - 26)
Vaccine type, n (%)		
Pfizer	0 (0.0)	6 (75.0)
Moderna	0 (0.0)	0 (0.0)
Mix of Pfizer and Moderna	0 (0.0)	2 (25.0)
**Anti-TNF treated IBD patients**		
Total, n	12	12
Female, n (%)	9 (75)	9 (75)
Age, y, median (IQR)	35 (28 - 47)	35.5 (28 - 47.5)
Age of IBD diagnosis, y, median (IQR)	22 (15 - 29)	22 (15 - 29)
BMI, kg/m^2, median (IQR)	24 (21 - 26)	24 (21 - 26)
Vaccine type, n (%)		
Pfizer	0 (0.0)	9 (75.0)
Moderna	0 (0.0)	3 (25.0)
Mix of Pfizer and Moderna	0 (0.0)	0 (0.0)
Type of infusion medication, n (%)		
Dosage and frequency, n (%)		
Infliximab	4 (33.3)	4 (33.3)
300mg q8w	1 (25.0)	1 (25.0)
500mg q4w	1 (25.0)	1 (25.0)
700mg q8w	1 (25.0)	1 (25.0)
3 vials of 100mg	1 (25.0)	1 (25.0)
Adalimumab	7 (58.3)	7 (58.3)
40mg q1w	3 (42.9)	3 (42.9)
40mg q2w	3 (42.9)	3 (42.9)
1 pen q1w	1 (14.3)	0 (0.0)
1 pen per 10 days	1 (8.3)	1 (14.3)
Golimumab	1 (8.3)	1 (8.3)
2 pens q2w	1 (100.0)	1 (100.0)
Disease state, n (%)		
Remission	9 (75.0)	10 (83.3)
Active disease	2 (16.7)	1 (8.3)
Unknown	1 (8.3)	1 (8.3)
**Anti-IL-12/23 treated IBD patients**		
Total, n	7	9
Female, n (%)	4 (57)	6 (67)
Age, y, median (IQR)	39 (24 - 57)	39 (29 - 57)
Age of IBD diagnosis, y, median (IQR)	14 (12 - 21)	14 (12 - 21)
BMI, kg/m^2, median (IQR)	22 (21 - 23)	22 (21 - 23)
Vaccine type, n (%)		
Pfizer	0 (0.0)	8 (88.9)
Moderna	0 (0.0)	1 (11.1)
Mix of Pfizer and Moderna	0 (0.0)	0 (0.0)
Type of infusion medication, n (%)		
Dosage and frequency, n (%)		
Ustekinumab	7 (100.0)	9 (100.0)
90mg q4w	3 (42.9)	5 (55.6)
90mg q6w	1 (14.3)	1 (11.1)
90mg q8w	2 (28.6)	2 (22.2)
1 syringe q8w	1 (14.3)	1 (11.1)
Disease state, n (%)		
Remission	5 (71.4)	8 (88.9)
Active disease	1 (14.3)	1 (11.1)
Unknown	1 (14.3)	0 (0.0)

Abbreviations: BMI: Body Mass Index; IBD: Inflammatory bowel disease; IL: Interleukin; IQR: Inter-quartile range; kg: Kilograms; m: Meters; Mos: Months; N/A: Sample not assessed; PD: Post dose; T: Timepoint; TNF: Tumor necrosis factor; Wk: Weeks; q(#w): every # weeks

Supplemental Table 2. Patient demographics: anti-Wuhan Spike IgG and IgM levels

Timepoint	T2: 2-4 WkPD1	T3: 2-4 WkPD2	T5: 2-4 WkPD3	T7: 2-4 WkPD4
**Healthy controls**				
Total, n	10	10	4	N/A
Female, n (%)	4 (40)	4 (40)	2 (50)	N/A
Age, y, median (IQR)	30.5 (26 - 37)	30.5 (26 - 37)	32 (26 - 42.5)	N/A
BMI, kg/m^2, median (IQR)	25 (23 - 27)	25 (24 - 28)	25 (23 - 28)	N/A
Vaccine type, n (%)				
Pfizer	9 (90.0)	8 (80.0)	3 (75.0)	N/A
Moderna	0 (0.0)	0 (0.0)	0 (0.0)	N/A
Mix of Pfizer and Moderna	1 (10.0)	2 (20.0)	1 (25.0)	N/A
**Anti-TNF treated IBD patients**				
Total, n	15	16	12	3
Female, n (%)	10 (67)	10 (62)	8 (67)	2 (67)
Age, y, median (IQR)	36 (29 - 46)	35.5 (29 - 44)	34.5 (29.5 - 42)	47 (31 - 67)
Age of IBD diagnosis, y, median (IQR)	23 (14 - 30)	20.5 (12 - 29)	23 (14 - 29)	28 (23 - 48)
BMI, kg/m^2, median (IQR)	24 (23 - 28)	24 (23 - 27)	23 (22 - 26)	30 (21 - 31)
Vaccine type, n (%)				
Pfizer	11 (73.3)	12 (75.0)	9 (75.0)	1 (33.3)
Moderna	4 (26.7)	4 (25.0)	2 (16.7)	1 (33.3)
Mix of Pfizer and Moderna	0 (0.0)	0 (0.0)	1 (8.3)	1 (33.3)
Type of infusion medication, n (%)				
Dosage and frequency, n (%)				
Infliximab	8 (53.3)	7 (58.3)		2 (66.7)
Adalimumab	7 (46.7)	7 (43.8)	5 (41.7)	1 (33.3)
Golimumab	0 (0.0)	0 (0.0)	0 (0.0)	0 (0.0)
Disease state, n (%)				
Remission	10 (66.7)	13 (81.2)	11 (91.7)	3 (100.0)
Active disease	1 (6.7)	1 (6.2)	1 (8.3)	0 (0.0)
Unknown	4 (26.7)	2 (12.5)	0 (0.0)	0 (0.0)
**Anti-IL-12/23 treated IBD patients**				
Total, n	12	13	7	5
Female, n (%)	8 (67)	9 (69)	4 (57)	2 (40)
Age, y, median (IQR)	34.5 (27.5 - 46.5)	35 (28 - 45)	39 (35 - 48)	35 (26 - 49)
Age of IBD diagnosis, y, median (IQR)	15 (12 - 23)	15 (13 - 27)	19 (15 - 29)	14 (13 - 15)
BMI, kg/m^2, median (IQR)	22 (20 - 23)	22 (20 - 23)	23 (20 - 26)	24 (22 - 24)
Vaccine type, n (%)				
Pfizer	11 (91.7)	12 (92.3)	7 (100.0)	4 (80.0)
Moderna	1 (8.3)	1 (7.7)	0 (0.0)	0 (0.0)
Mix of Pfizer and Moderna	0 (0.0)	0 (0.0)	0 (0.0)	1 (20.0)
Type of infusion medication, n (%)				
Dosage and frequency, n (%)				
Ustekinumab	12 (100.0)	13 (100.0)	7 (100.0)	5 (100.0)
Disease state, n (%)				
Remission	9 (75.0)	10 (76.9)	7 (100.0)	5 (100.0)
Active disease	2 (16.7)	1 (7.7)	0 (0.0)	0 (0.0)
Unknown	1 (8.3)	2 (15.4)	0 (0.0)	0 (0.0)

Abbreviations: BMI: Body Mass Index; IBD: Inflammatory bowel disease; IL: Interleukin; IQR: Inter-quartile range; kg: Kilograms; m: Meters; Mo: Months; N/A: Sample not assessed; PD: Post dose; T: Timepoint; TNF: Tumor necrosis factor; Wk: Weeks

Supplemental Table 3. Patient demographics: anti-Wuhan Spike IgG avidity measurement

Timepoint	T2: 2-4 WkPD1	T3: 2-4 WkPD2	T4: 3-4 MoPD2	T5: 2-4 WkPD3	T6: 3-4 MoPD4	T7: 2-4 WkPD4	T8: 2-4 WkPD4
**Healthy Controls**							
Total[A], n	14	14	10	5	6	N/A	N/A
Included in longitudinal analyses,[B] n	12	12	10	5	6	N/A	N/A
Female, n (%)	6 (50)	6 (50)	4 (40)	3 (60)	3 (50)	N/A	N/A
Age, y, median (IQR)	26.5 (25.5 - 36.5)	27 (25.5 - 37)	27 (25 - 37)	27 (25 - 37)	31 (26 - 37)	N/A	N/A
BMI, kg/m^2, median (IQR)	24 (22 - 27)	24 (22 - 27)	24 (22 - 27)	23 (23 - 28)	24 (22 - 27)	N/A	N/A
Vaccine type, n (%)							
Pfizer	11 (91.7)	10 (83.3)	8 (80.0)	4 (80.0)	4 (66.7)	N/A	N/A
Moderna	0 (0.0)	0 (0.0)	0 (0.0)	0 (0.0)	0 (0.0)	N/A	N/A
Mix of Pfizer and Moderna	1 (8.3)	2 (16.7)	2 (20.0)	1 (20.0)	2 (33.3)	N/A	N/A
**Anti-TNF treated IBD patients**							
Total[A], n	15	16	12	12	8	3	N/A
Included in longitudinal analyses,[B] n	12	15	12	12	8	3	N/A
Female, n (%)	9 (75)	9 (60)	7 (58)	8 (67)	4 (50)	2 (67)	N/A
Age, y, median (IQR)	33.5 (28.5 - 44)	35 (29 - 42)	32.5 (28 - 39)	34.5 (29.5 - 42)	35 (30.5 - 39.5)	47 (31 - 67)	N/A
Age of IBD diagnosis, y, median (IQR)	33.5 (28.5 - 44)	35 (29 - 42)	32.5 (28 - 39)	34.5 (29.5 - 42)	35 (30.5 - 39.5)	47 (31 - 67)	N/A
BMI, kg/m^2, median (IQR)	24 (22 - 27)	24 (23 - 28)	25 (22 - 27)	23 (22 - 26)	24 (22 - 27)	30 (21 - 31)	N/A
Vaccine type, n (%)							
Pfizer	9 (75.0)	12 (80.0)	9 (75.0)	9 (75.0)	5 (62.5)	1 (33.3)	N/A
Moderna	3 (25.0)	3 (20.0)	3 (25.0)	2 (16.7)	2 (25.0)	1 (33.3)	N/A
Mix of Pfizer and Moderna	0 (0.0)	0 (0.0)	0 (0.0)	1 (8.3)	1 (12.5)	1 (33.3)	N/A
Type of infusion medication, n (%)							
Infliximab	6 (50.0)	9 (60.0)	8 (66.7)	7 (58.3)	6 (75.0)	2 (66.7)	N/A
Adalimumab	6 (50.0)	6 (40.0)	4 (33.3)	5 (41.7)	2 (25.0)	1 (33.3)	N/A
Disease state, n (%)							
Remission	8 (66.7)	12 (80.0)	9 (75.0)	11 (91.7)	8 (100.0)	3 (100.0)	N/A
Active disease	1 (8.3)	1 (6.7)	2 (16.7)	1 (8.3)	0 (0.0)	0 (0.0)	N/A
Unknown	3 (25.0)	2 (13.3)	1 (8.3)	0 (0.0)	0 (0.0)	0 (0.0)	N/A
**Anti-IL-12/23 treated IBD patients**							
Total[A], n	14	13	10	8	8	6	5
Included in longitudinal analyses,[B] n	12	12	10	8	8	6	5
Female, n (%)	8 (67)	8 (67)	7 (70)	5 (62)	6 (75)	3 (50)	2 (40)
Age, y, median (IQR)	37 (29 - 46.5)	37 (29 - 46.5)	41 (35 - 48)	41 (35 - 47)	38 (28.5 - 52)	40.5 (28 - 49)	35 (28 - 49)
Age of IBD diagnosis, y, median (IQR)	37 (29 - 46.5)	37 (29 - 46.5)	41 (35 - 48)	41 (35 - 47)	38 (28.5 - 52)	40.5 (28 - 49)	35 (28 - 49)
BMI, kg/m^2, median (IQR)	22 (20 - 23)	22 (20 - 24)	23 (21 - 26)	24 (21 - 25)	24 (21 - 28)	23 (21 - 24)	24 (20 - 24)
Vaccine type, n (%)							
Pfizer	12 (100.0)	12 (100.0)	10 (100.0)	8 (100.0)	8 (100.0)	6 (100.0)	5 (100.0)
Moderna	0 (0.0)	0 (0.0)	0 (0.0)	0 (0.0)	0 (0.0)	0 (0.0)	0 (0.0)
Mix of Pfizer and Moderna	0 (0.0)	0 (0.0)	0 (0.0)	0 (0.0)	0 (0.0)	0 (0.0)	0 (0.0)
Type of infusion medication, n (%)							
Ustekinumab	12 (100.0)	12 (100.0)	10 (100.0)	8 (100.0)	8 (100.0)	6 (100.0)	5 (100.0)
Disease state, n (%)							
Remission	9 (75.0)	9 (75.0)	8 (80.0)	8 (100.0)	8 (100.0)	6 (100.0)	4 (80.0)
Active disease	2 (16.7)	1 (8.3)	1 (10.0)	0 (0.0)	0 (0.0)	0 (0.0)	0 (0.0)
Unknown	1 (8.3)	2 (16.7)	1 (10.0)	0 (0.0)	0 (0.0)	0 (0.0)	1 (20.0)

[A] Total number of participant samples for which anti-Spike IgG avidity assessments were conducted (Figure 7D-E).
[B] Participants were only included in longitudinal analyses (Figure 7A) if they had samples from at least two different timepoints available for avidity assessments. Abbreviations: BMI: Body Mass Index; IBD: Inflammatory bowel disease; IL: Interleukin; IQR: Inter-quartile range; kg: Kilograms; m: Meters; Mo: Months; N/A: Sample not assessed; PD: Post dose; T: Timepoint; TNF: Tumor necrosis factor; Wk: Weeks; q(#w): every # weeks

Supplemental Table 4. Anti-Wuhan Spike IgG avidity measurements

Anti-Wuhan Spike IgG avidity measurements<br>Comparisons between timepoints[A]			
	**T2: 2-4 WkPD1 vs T3: 2-4 WkPD2**	**T3: 2-4 WkPD2 vs T5: 2-4 WkPD3**	**T5: 2-4 WkPD3 vs T7: 2-4 WkPD4**
**Healthy controls**	p = 0.000	p = 0.040	N/A
**Anti-TNF treated IBD**	p = 0.000	p = 0.000	p = 0.428
**Anti-IL-12/23 treated IBD**	p = 0.000	p = 0.019	p = 0.728

[A] Multivariate longitudinal regression analyses controlled for age, sex, BMI, vaccine type and IgG concentration, with an interaction between timepoint and study group. Estimated margins (mean IgG avidity) were compared between timepoints (T) (as defined in Figure 1A) within a study group using the “lincom” post-estimation command in STATA 18, with p-values displayed in this table.
Abbreviations: IBD: Inflammatory bowel disease; IL: Interleukin; N/A: Sample not assessed; PD: Post dose; T: Timepoint; TNF: Tumor necrosis factor; Wk: Weeks

Supplemental Table 5. Calculation of relative avidity index, fractional relative avidity index and total relative avidity index

**NH_4SCN Concentration (molar [M])**	**2M**	**1M**	**0.5M**	**0M**	**NA[D]**
**Area under the curve[A]**	A_2	A_1	$A_{0.5}$	A_0	NA
**Relative Avidity Index (RAI) (%)[B]**	RAI_2= A_2/A_0*100	RAI_1= A_1/A_0*100	$RAI_{0.5}$= $A_{0.5}/A_0$*100	NA	NA
**Fractional (F) RAI (%)[C]** <br>**Associated categorical avidity level**	F RAI_2= RAI_2 <br>high avidity	F RAI_1= RAI_1-RAI_2 <br>medium avidity	F $RAI_{0.5}$= $RAI_{0.5}$-RAI_1 <br>low avidity	NA	F $RAI_{<0.5}$= 100-$RAI_{0.5}$ <br>very low avidity
**Total RAI (AU)**	2 * F RAI_2 + 1 * F RAI_1 + 0.5 * F $RAI_{0.5}$ + 0.25 * F $RAI_{<0.5}$				

[A] Area under the plasma dilution curve (AUC) with and without treatment of chaotropic reagent. For analyses of the avidity of anti-BA.5 RBD IgG, samples with AUC values ≤ 0.2 were excluded.
T RAI: Total relative avidity index; weighted sum of the proportion of Abs present at each fraction of chaotropic reagent.
[B] RAI values < 0.5% were assigned a value of 0.5 for statistical purposes.
[C] F RAI values < 0% were assigned a value of 0 for statistical purposes. F RAI values translate to the proportion of IgG that fall into the associated categorial avidity level.
[D] This column calculates the F RAI of anti-Spike or anti-BA.5 RBD IgG Abs eluted by the lowest concentration (0.5M) of ammonium thiocyanate. F $RAI_{<0.5}$ < 0% were assigned a value of 0 for statistical purposes.
Abbreviations: AU: Avidity Unit; F: Fractional; NH_4SCN: Ammonium thiocyanate; RAI: Relative avidity index; T: Total

Supplemental Table 6. Flow cytometry panel used to sort cells for single-cell RNA-Seq

Target	Fluorophore	Clone	Vendor and Catalogue
Live/Dead	eF506	N/A	Invitrogen eBioscience<br>Cat: 65-0866-14
CD3	AF700	UCHT1	Biolegend<br>Cat: 300424
CD19	BV786	SJ2SC1	BD Horizon<br>Cat: 563325
CD27	BB700	MT271	BD Horizon<br>Cat: 566449
IgD	BV421	IA6-2	Biolegend<br>Cat: 348226
TotalSeq-C Hashtags Antibodies #1-12	N/A	N/A	Biolegend<br>Cat: 394661, 394663, 394665, 394667, 394669, 394671, 394673, 394675, 394677, 394679, 394683, 328941
Wuhan Spike-PE tetramer	PE	N/A	Refer to Methods
Horizon Brilliant Buffer	N/A	N/A	BD Horizon<br>Cat: 563794

Supplemental Table 7: Flow cytometry panel used to detect Wuhan SARS-CoV-2 Spike-specific memory B cells

Target	Fluorophore	Clone	Vendor and Catalogue
Live/Dead	eF506	N/A	Invitrogen eBioscience<br>Cat: 65-0866-14
CD3	AF700	UCHT1	Biolegend<br>Cat: 300424
CD19	BV786	SJ2SC1	BD Horizon<br>Cat: 563325
CD20	BUV496	2H7	Invitrogen eBioscience<br>Cat: 364-0209-42
CD21	BV605	1048	BD OptiBuild<br>Cat: 742761
CD27	BB700	MT271	BD Horizon<br>Cat: 566449
CD23	BUV805	M-L233	BD OptiBuild<br>Cat: 742035
CD38	BV650	HB-7	Biolegend<br>Cat: 356619
CD1c	BUV737	F10/21A3	BD OptiBuild<br>Cat: 742035
CD11c	FITC	S-HCL-3	Biolegend<br>Cat: 301604
IgD	BV421	IA6-2	Biolegend<br>Cat: 348226
IgG	PE/Cy-7	M1310G05	Biolegend<br>Cat: 410722
IgM	BUV395	G20-127	BD Horizon<br>Cat: 563903
pan HLA-DR/DP/DQ	APC	Tu39	Biolegend<br>Cat: 361714
Wuhan Spike-PE tetramer	PE	N/A	Refer to Methods
Horizon Brilliant Buffer	N/A	N/A	BD Horizon<br>Cat: 563794
